# Role of PLK1/NUMB/NOTCH in epithelial-mesenchymal transition in human melanoma

**DOI:** 10.1038/s41698-023-00493-7

**Published:** 2024-01-06

**Authors:** Gagan Chhabra, Chandra K. Singh, Mary A. Ndiaye, Shengqin Su, Carl A. Shirley, Nihal Ahmad

**Affiliations:** 1 Department of Dermatology, University of Wisconsin, Madison, WI 53705 USA; 2 William S. Middleton Memorial Veterans Hospital, Madison, WI 53705 USA

**Keywords:** Cancer, Skin cancer, Melanoma

## Abstract

Polo-like kinase 1 (PLK1), a serine/threonine kinase, is overexpressed in melanoma and its expression has been associated with poor disease prognosis. PLK1 has been shown to interact with NUMB, a NOTCH antagonist. However, the exact role of PLK1, NUMB, and NOTCH signaling in epithelial-mesenchymal transition (EMT) in melanoma progression is unclear. In this study, Affymetrix microarray analysis was performed to determine differentially expressed genes following shRNA-mediated knockdown of PLK1 in human melanoma cells that showed significant modulations in EMT and metastasis-related genes. Using multiple PLK1-modulated melanoma cell lines, we found that PLK1 is involved in the regulation of cell migration, invasion, and EMT via its kinase activity and NOTCH activation. In vitro kinase assay and mass spectrometry analysis demonstrated a previously unknown PLK1 phosphorylation site (Ser413) on NUMB. Overexpression of non-phosphorylatable (S413A) and phosphomimetic (S413D) mutants of NUMB in melanoma cells implicated the involvement of NUMB-S413 phosphorylation in cell migration and invasion, which was independent of NOTCH activation. To determine the clinical relevance of these findings, immunohistochemistry was performed using melanoma tissue microarray, which indicated a strong positive correlation between PLK1 and N-cadherin, a protein required for successful EMT. These findings were supported by TCGA analysis, where expression of high PLK1 with low NUMB or high NOTCH or N-cadherin showed a significant decrease in survival of melanoma patients. Overall, these results suggest a potential role of PLK1 in EMT, migration, and invasion of melanoma cells. Our findings support the therapeutic targeting of PLK1, NUMB, and NOTCH for melanoma management.

## Introduction

Melanoma is an aggressive skin cancer that can disseminate from primary tumors and metastasize to multiple sites, including lymph nodes, lungs, liver, brain, and bone. Although the treatment landscape for advanced melanoma has improved dramatically with the approval of targeted and immunotherapy drugs, increasing rates of disease progression due to acquired resistance present a major challenge^1^. Therefore, a deeper understanding of melanoma progression and signaling mechanisms involved in metastasis is required for developing newer therapeutics for the effective management of this deadly neoplasm.

Mammalian polo-like kinase 1 (PLK1), an important serine/threonine kinase, has been shown to be a critical regulator of mitosis and cytokinesis. Dysregulation of PLK1 results in cell cycle aberrations that often incite cell proliferation (reviewed in^2^). In fact, several studies, including those from our laboratory, have shown that PLK1 is significantly overexpressed in several cancers, including melanoma (reviewed in^3^). In an earlier study from our lab, we have shown that PLK1 knockdown in A375 melanoma cells reduced growth and altered metabolic regulation^4^. We have also shown that targeted depletion of PLK1 through lentiviral shRNA or a small-molecule inhibitor resulted in a significant decrease in clonogenic survival, G2/M cell-cycle arrest, and apoptosis of human melanoma cells, suggesting that PLK1 might have functional relevance in melanoma development and/or progression^5^. In a previous study, we also identified an association between PLK1 and NUMB (an antagonist of NOTCH pathway) during the progression of mitosis in melanoma cells and proposed that loss of NUMB may contribute to tumorigenesis through dysregulation of PLK1 leading to chromosome abnormalities and aneuploidy^6^. However, the exact role and significance of PLK1, NUMB, and NOTCH in melanoma remains to be determined. NUMB is an evolutionarily conserved protein that plays critical roles in cell fate determination, cell polarity maintenance, and cell migration. In a very recent study, NUMB expression was shown to be downregulated in metastatic melanoma cells. Further, the knockdown of NUMB significantly increased the invasion potential of melanoma cells in vitro and in the lungs of a mouse model in vivo by modulating the NOTCH target gene CCNE^7^. Interestingly, the NUMB gene is alternatively spliced to generate six functionally distinct transcript variants (isoforms), out of which four transcript variants NUMB1-651 aa; NUMB2-603 aa; NUMB3-640 aa; NUMB4-592 aa, are the most prevalent^8^. These NUMB isoforms have been shown to play different roles in different cancers^9–11^. However, the role and functional significance of NUMB isoforms in melanoma is not clear. In this study, we focused on NUMB isoform 1 (NUMB1; also known as transcript variant 1) to further expand our previously published research on this variant and its interactions with PLK1^6^.

Epithelial-mesenchymal transition (EMT) is an important mechanism of tumor progression and metastasis that causes a loss of epithelial cell characteristics (e.g. cell–cell junctions, apicobasal polarity of the cell, and cobblestone morphology) and acquisition of mesenchymal characteristics (e.g. increased cell-matrix adhesions and motility, and fibroblast-like cell morphology)^12^. Further, EMT results in increased cell migration and invasion, which allows cancer cells to invade the surrounding vasculature, leading to tumor dissemination and metastases^13^. PLK1 has been shown as a key regulator of EMT and cell motility in prostate cancer^14^, and a limited number of recent studies have suggested that PLK1 could play a role in EMT^15,16^. Further, it has also been suggested that NUMB is involved in cancer progression with a critical role in EMT^9,17–19^. However, direct evidence supporting the involvement of PLK1 and NUMB in EMT process in melanoma is lacking. Thus, we hypothesized that PLK1 has a role in cellular invasion and metastasis by inducing EMT in melanoma cells and PLK1 phosphorylation of NUMB may be an important step in cellular reprogramming causing EMT via activation of the NOTCH pathway in melanoma cells.

In this study, we determined the functional significance of PLK1 in EMT in melanoma cells by implementing PLK1 gain-of-function and loss-of-function approaches. Our data provide evidence that PLK1 plays an important role in cell migration, invasion, and EMT in melanoma. Further, we have identified a previously unknown PLK1 phosphorylation site on NUMB. In addition, we demonstrated that PLK1 kinase activity leads to activation of NOTCH signaling and promotes EMT. To determine the clinical relevance of these findings, we conducted immunohistochemistry analysis using melanoma tissue microarray (TMA) and found a strong positive correlation of PLK1 with N-cadherin, a protein required for successful EMT. These findings were supported by the results obtained from TCGA melanoma data analysis, where high PLK1 expression with low NUMB or high NOTCH or high N-cadherin was associated with a significant decrease in survival of melanoma patients. Altogether, our study suggests that targeting PLK1, NUMB, and NOTCH may be a potential therapeutic strategy in inhibiting melanoma progression.

## Results

### Affymetrix microarray analysis shows modulation in EMT and metastasis-related genes in PLK1-knockdown melanoma cells

**Fig. 1:**
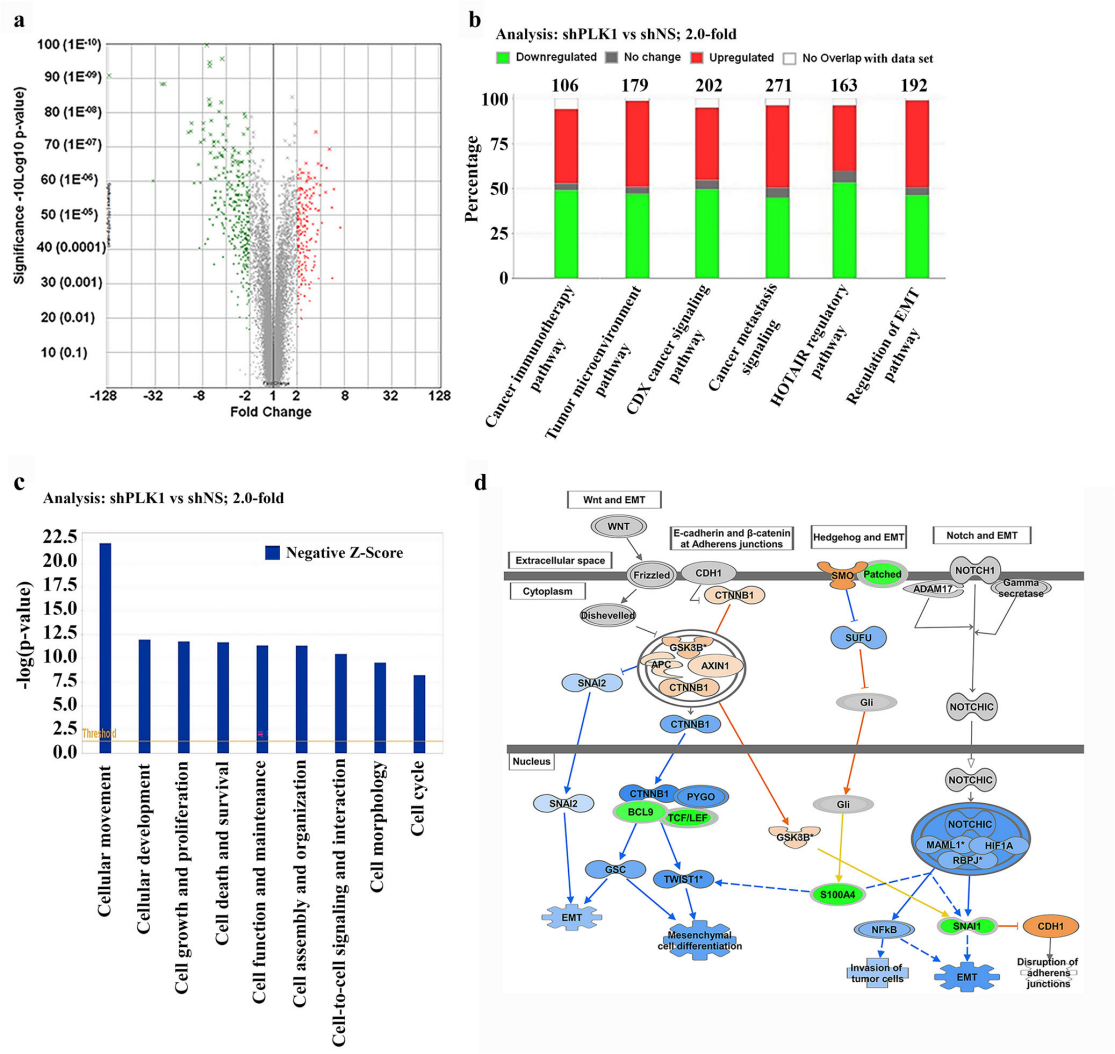
Affymetrix Human Transcriptome Array 2.0 and Ingenuity Pathway Analysis (IPA) with PLK1 knockdown in melanoma cells. **a** Volcano plot showing differentially modulated genes representing the logarithm of fold change (log2) on the x-axis and −log10 of the p-value on the y-axis. Green and red dots correspond to significantly downregulated or upregulated genes. **b** Cancer-related top canonical pathways generated through IPA analysis. The stacked bar chart indicates percentage of genes that were upregulated (red), downregulated (green), no change (gray), or not overlapped with the dataset (white) in each canonical pathway. The total number of genes in specific canonical pathways is mentioned at the top of each bar. **c** Top cellular functions identified by IPA. Negative z-score indicates a pathway with genes under the state of inhibition (blue bars). **d** Expanded view of regulation of EMT pathway showing modulated genes affecting cell migration. Green symbols correspond to ‘downregulated’, blue for ‘inhibited’, and orange for ‘increased’, respectively.

To explore the PLK1-associated mechanisms and to identify genes affected by PLK1 signaling in melanoma, we used an Affymetrix microarray to compare the shRNA-mediated PLK1 knockdown in A375 melanoma cell line (A375 shPLK1) with its nonsense control cell line (A375 shNS). A list of genes that were found to be significantly upregulated and downregulated after PLK1-knockdown (*P*  <  0.05 and fold change >2.0) has been provided in Supplementary Table 1. This data is represented in a volcano plot (Fig. 1a). Canonical pathway analysis was performed using Ingenuity Pathway Analysis (IPA) for genes that were significantly modulated, and several of the top significantly modulated cancer-associated pathways are shown in Fig. 1b. Interestingly, three of these identified pathways were, i) cancer metastasis signaling, ii) HOTAIR regulatory pathway, and iii) regulation of EMT pathway, suggesting a potential role of PLK1 in EMT and melanoma metastasis. Additionally, IPA was used to identify key biological processes influenced by PLK1 knockdown and found inhibition of several key cellular functions including cellular movement as a top hit with others such as cellular development, cell-to-cell signaling and interaction, and cell morphology (Fig. 1c). A network diagram was created via IPA to show the potential EMT-related signaling molecules that may be affected by PLK1 knockdown. One of the molecules identified in this analysis is SNAIL, an EMT transcription factor, which was found to be significantly downregulated by PLK1 knockdown, likely due to upstream NOTCH signaling (Fig. 1d). Next, IPA was used to understand the cumulative actions of genes identified in response to PLK1 inhibition, and this analysis predicted the inhibition of migration of cancer cells (Supplementary Fig. 1a). Additionally, heatmap data predicted the inhibition (indicated by blue color) of cellular movement-related processes and functions in response to PLK1 knockdown (Supplementary Fig. 1b). Overall, our Affymetrix microarray data indicated that PLK1 might be an important signaling molecule involved in EMT and metastasis-related functions in melanoma.

### PLK1 knockdown inhibits melanoma cell proliferation, migration, invasion and EMT markers

**Fig. 2:**
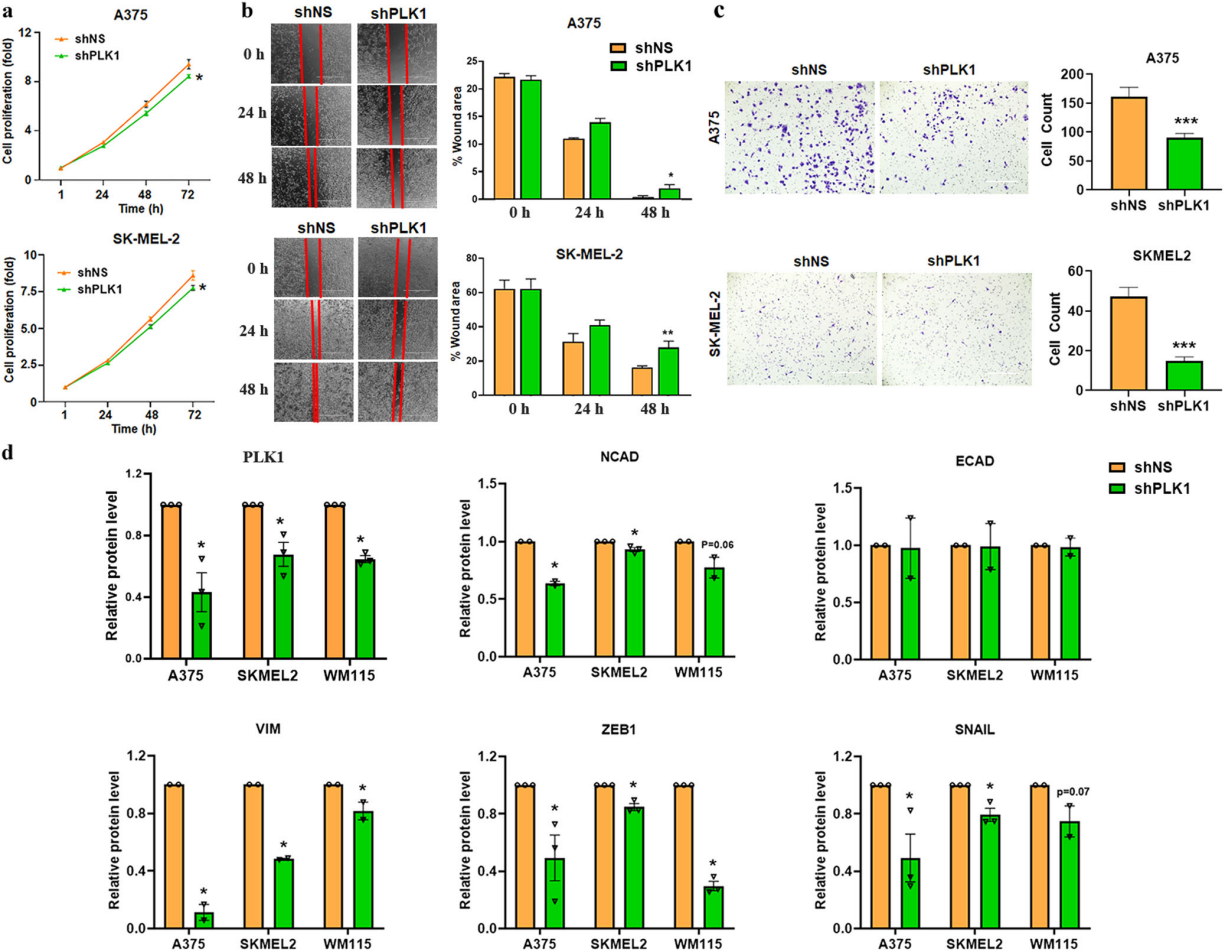
Cell proliferation, migration, invasion, and EMT-related proteins affected by PLK1 knockdown in melanoma cells. **a** Cell proliferation assay was performed using RealTime-Glo MT Cell Viability Assay (Promega) in melanoma cells up to 72 h. **b** Cell migration was analyzed by wound healing assay in melanoma cells at 0, 24 and 48 h post-wound creation. **c** Cell invasion was analyzed by Matrigel invasion chambers using melanoma cells at 24 h. For **a**–**c**, the quantitative data are presented as mean ± SEM with statistical significance compared to shNS control (*p < 0.05; **p < 0.01; ***p < 0.001) in ≥2 biological replicates with ≥3 technical replicates (2-3 images per replicate for migration and invasion assay). Scale Bar=400 µm. **d** Simple Western immunoblot analysis showing EMT proteins in melanoma cells. The relative protein levels normalized to total protein are presented as mean ± SEM with statistical significance compared to shNS control (*p < 0.05) in ≥2 biological replicates of each cell line. Statistical significance was determined via multiple t-tests using the Holm–Sidak method.

To further experimentally validate the effects of PLK1 on the cellular migration of melanoma cells, we performed wound healing and Matrigel invasion assays. Inhibition of endogenous PLK1 by shRNA-mediated knockdown decreased cell proliferation (Fig. 2a), migration (Fig. 2b), and invasion (Fig. 2c) of shPLK1 melanoma cells compared to the control cells. A significant difference in cell proliferation was observed at 72 h but not at 24-48 h. Hence, the observed decreased migratory (at 48 h) and invasive (at 24 h) behavior appears to be due to PLK1 knockdown rather than due to a decrease in the proliferation of melanoma cells. To confirm the effects of PLK1 on EMT, we performed a Simple Western analysis of key EMT markers in shNS and shPLK1 harboring A375, WM115, and SK-MEL-2 melanoma cells. We found significant downregulation of mesenchymal markers such as N-cadherin, and Vimentin, and transcriptional inducers of EMT like SNAIL and ZEB1 in shPLK1 cells, as compared to shNS control (Fig. 2d), indicating a potential reversal of EMT. Additionally, we performed immunofluorescence analysis of EMT markers E-cadherin and N-cadherin after PLK1 knockdown in melanoma cells. We found that inhibition of PLK1 upregulates E-cadherin and significantly downregulates N-cadherin (Supplementary Fig. 2) in SK-MEL-2 melanoma cells. These results correspond with and support our Affymetrix microarray data.

### PLK1 overexpression and kinase activity promote melanoma cell proliferation, migration, invasion and EMT

**Fig. 3:**
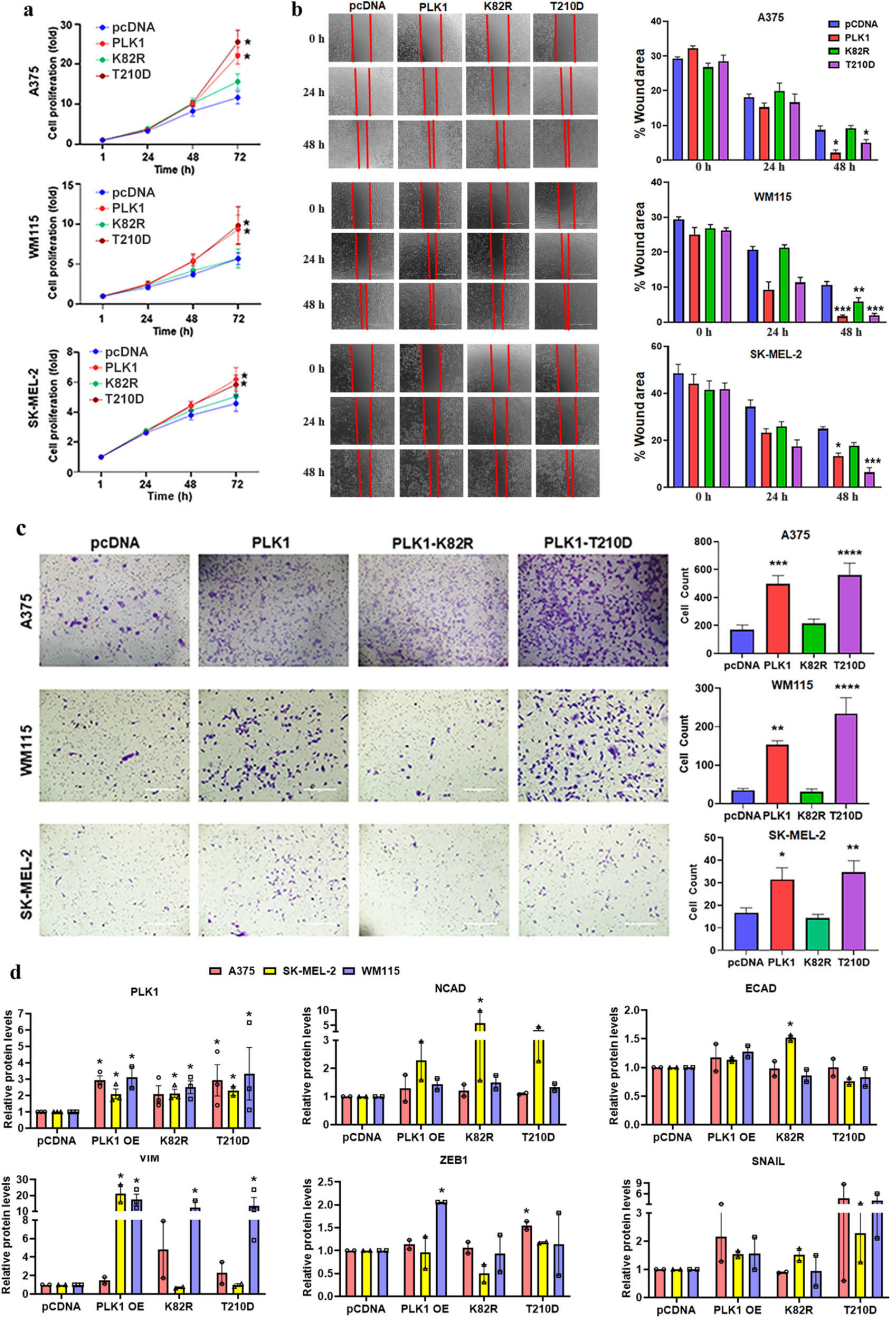
Cell proliferation, migration, invasion, and EMT-related proteins affected by PLK1 wild-type and mutant kinase overexpression activity in melanoma cells. **a** Cell proliferation assay was performed using RealTime-Glo MT Cell Viability Assay (Promega) in PLK1-modulated melanoma cells (PLK1-wild type; K82R- constitutively inactive; T210D- constitutively active) till 72 h. **b** Cell migration was analyzed by wound healing assay in PLK1-modulated melanoma cells at 0, 24, and 48 h post-wound creation. **c** Cell invasion was analyzed by Matrigel invasion chambers using PLK1-modulated melanoma cells at 24 h. For **a**–**c**, the quantitative data are presented as mean ± SEM with statistical significance compared to empty vector (pcDNA) control (*p < 0.05; **p < 0.01 ***p < 0.001; ****p < 0.0001) in ≥2 biological replicates with ≥3 technical replicates (2-3 images per replicate for migration and invasion assay). Scale Bar=400 µm. **d** Simple Western immunoblot analysis showing expression of EMT-related proteins in PLK1-modulated melanoma cells. The relative protein levels normalized to total protein are presented as mean ± SEM with statistical significance compared to shNS control (*p < 0.05) in ≥2 biological replicates of each cell line. Statistical significance was determined using one- or two-way ANOVA followed by Fisher’s LSD tests.

In the next series of experiments, we determined the effects of forced overexpression of PLK1 on melanoma cell proliferation, migration, invasion and EMT markers. PLK1 overexpression (PLK1 OE) in A375, WM115 and SK-MEL-2 melanoma cells enhanced cell proliferation (Fig. 3a), migration (Fig. 3b) and invasion (Fig. 3c) compared to the empty vector control (pcDNA) cells. Simple Western analysis was performed to confirm PLK1 overexpression in melanoma cells and its effect on EMT markers, which showed an increasing trend in the expression of mesenchymal markers, such as N-cadherin and Vimentin, as well as transcription factors ZEB1 and SNAIL (Fig. 3d). To determine whether the ability of PLK1 to promote melanoma cell migration, invasion, and EMT was dependent on its phosphorylation activity, we overexpressed constitutively kinase-active (T210D) and kinase-inactive (K82R) PLK1 in A375, WM115, and SK-MEL-2 melanoma cells. Melanoma cells with constitutively kinase-active PLK1 showed higher proliferation, migration, and invasion potential (Fig. 3a-c), as well as modulation in EMT markers compared to the kinase-inactive PLK1 expressing cells (Fig. 3d). Interestingly, a significant difference in cell proliferation was observed at 72 h but not at 24-48 h. Therefore, the observed increased migratory (at 48 h) and invasive (at 24 h) behavior appears to be due to PLK1 manipulation rather than due to an increase in the proliferation of melanoma cells. These results suggest that PLK1 expression as well as kinase activity are an important factor in promoting EMT in melanoma cells.

### PLK1 phosphorylates NUMB at Ser413 and modulates the EMT process in melanoma

**Fig. 4:**
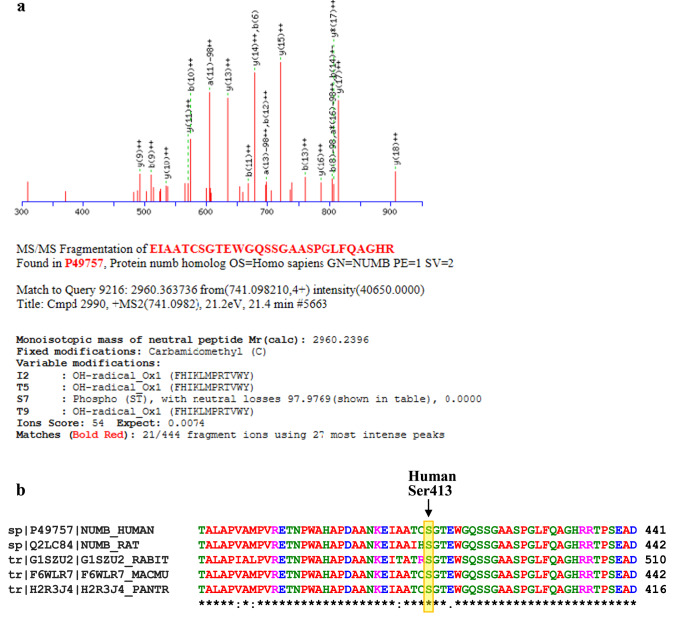
Mass spectrometry analysis to identify a previously unknown PLK1 phosphorylation site on NUMB. **a** Bottom-up mass spectrometry analysis after in-vitro kinase assay followed by an error-tolerant search revealed NUMB peptide with phosphorylated serines at positions S413. **b** Clustal Omega multiple sequence alignment for NUMB protein sequences showed conserved Ser413 among different vertebrate species.

**Fig. 5:**
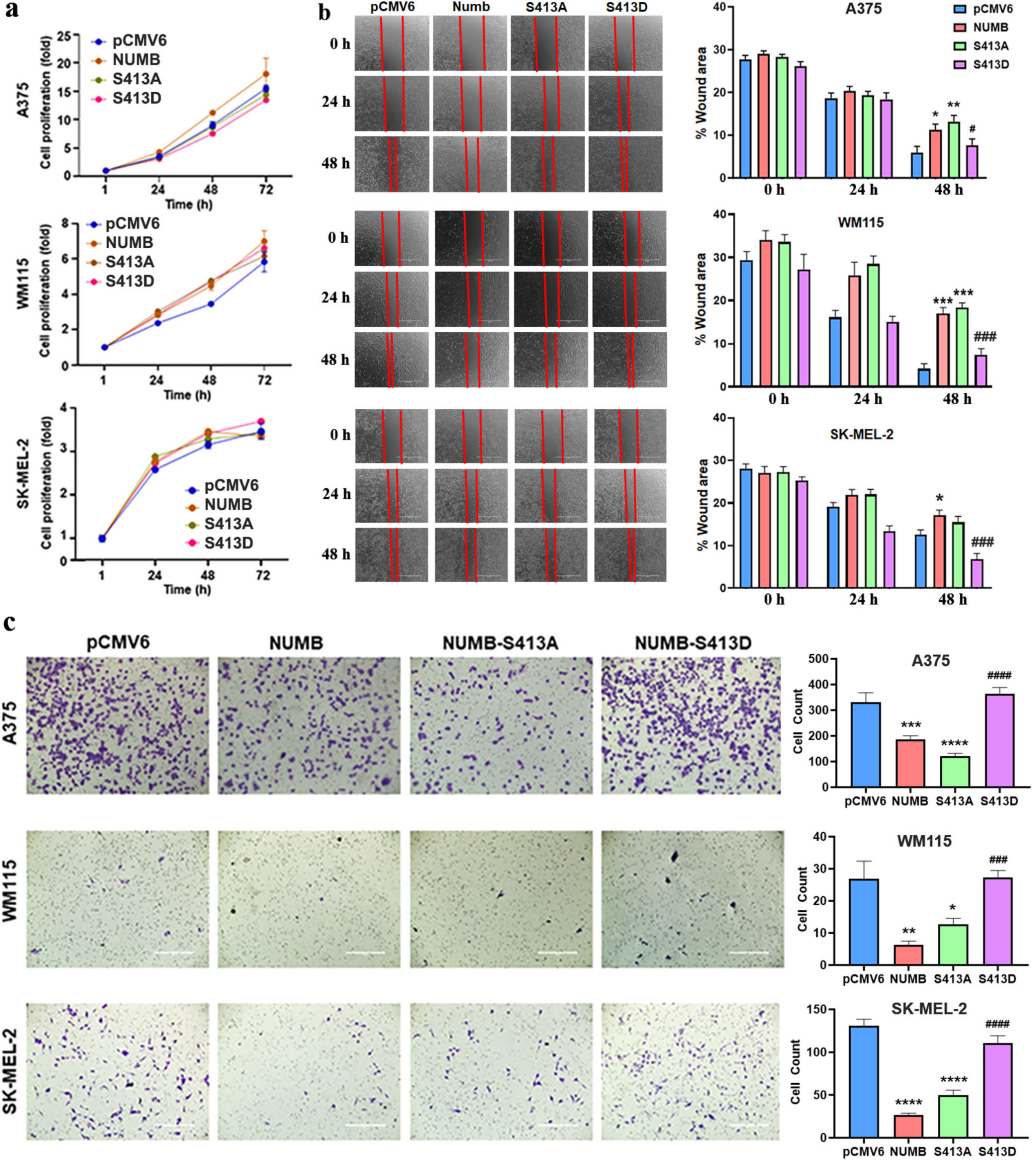
Cell proliferation, migration, and invasion affected by NUMB modulation in melanoma cells. **a** Cell proliferation assay was performed using RealTime-Glo MT Cell Viability Assay (Promega) in NUMB-overexpressing (NUMB-wild type; S413A-non-phosphorylatable; S413D-phosphomimetic) melanoma cells till 72 h. **b** Cell migration was analyzed by wound healing assay in NUMB-modulated melanoma cells at 0, 24 and 48 h post-wound creation. **c** Cell invasion was analyzed by Matrigel invasion chambers using NUMB-modulated melanoma cells at 24 h. The quantitative data are presented as mean ± SEM with statistical significance compared to empty vector (pCMV6) control (**p < 0.01; ***p < 0.001; ****p < 0.0001) or compared to NUMB overexpression (^**#**^*p* < 0.05; ^**###**^*p* < 0.001; ^**####**^*p* < 0.0001 in ≥2 biological replicates with ≥3 technical replicates and 2-3 images per replicate. Scale Bar=400 µm. Statistical significance was determined using one- or two-way ANOVA followed by Fisher’s LSD tests.

Previously, we have shown that PLK1 interacts with NUMB^6^, which is an evolutionarily conserved protein involved in cell fate determination, polarity, and migration. Since NUMB has also been linked with EMT regulation and is known to directly interact with PLK1, we attempted to determine the putative PLK1 phosphorylation site(s) of NUMB. To demonstrate NUMB phosphorylation by PLK1, we initially performed an in-vitro kinase assay using recombinant purified PLK1 and NUMB (transcript variant 1) proteins followed by bottom-up mass spectrometry after in-solution digestion with trypsin. An error-tolerant search of all significant protein hits revealed a NUMB peptide with phosphorylated serine at position S413 (Fig. 4a). Interestingly, Clustal Omega multiple sequence alignment for NUMB protein sequences showed conserved S413 among different vertebrate species (Fig. 4b). Moreover, we obtained an antibody against phospho-S413 (p-S413) NUMB peptide, which was able to detect phosphorylated NUMB protein by immunoblot analysis after in-vitro kinase assay using purified PLK1 and NUMB. This p-S413 site on NUMB was further confirmed using site-directed mutagenesis to change the serine at the 413 site to alanine to prevent phosphorylation. Immunoblot analysis after in-vitro kinase assay using PLK1 and mutated S413A-NUMB protein showed that phosphorylation was blocked in the mutant NUMB, confirming that this is a PLK1 phosphorylation site (Supplementary Fig. 3). In addition, we conducted cell proliferation, migration and invasion assays utilizing WT NUMB overexpression, S413A-NUMB (non-phosphorylatable) and S413D-NUMB (phosphomimetic) clones of A375, WM115, and SK-MEL-2 melanoma cells. The results suggested that NUMB overexpression, S413A, and S413D mutations did not affect cell proliferation of melanoma cells (Fig. 5a). Interestingly, forced overexpression of NUMB inhibited invasion of melanoma cells significantly when compared to empty vector control, and the S413D phosphomimetic mutant significantly rescued NUMB-inhibited invasion when compared to NUMB overexpressing cells. These results were consistent in all three melanoma cell lines tested. Moreover, similar to NUMB overexpression, non-phosphorylatable S413A-NUMB mutation showed decreased migration and invasion (Fig. 5b, c). Further, we analyzed protein levels of PLK1, NICD (NOTCH intracellular domain), and EMT-related markers in NUMB-modulated melanoma cells. We found that NUMB overexpression and mutations (S413A/S413D) did not significantly change PLK1 and NICD protein levels. However, we found changes in EMT-related proteins, which were not consistent in these three melanoma cell lines. These results have been included in Supplementary Fig. 4.

**Fig. 6:**
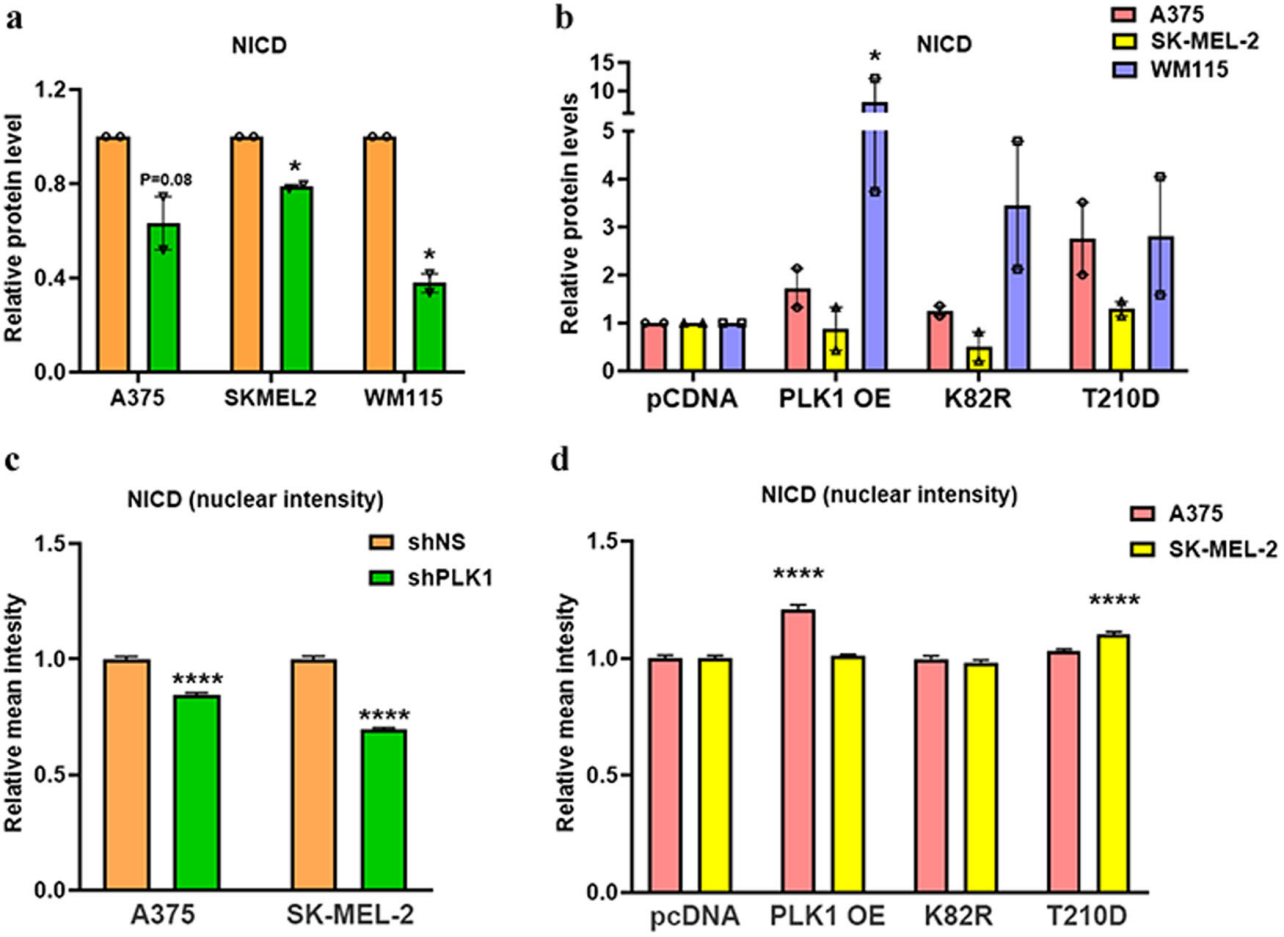
Simple Western and immunofluorescence analyses of Notch intracellular domain (NICD) in PLK1-modulated melanoma cells. **a**, **b** Simple Western immunoblot analysis showing expression of NICD in PLK1-modulated melanoma cells. The relative protein levels normalized to total protein are presented as bar graphs. **c**, **d** Relative mean intensity analyzed by immunofluorescence analysis for nuclear localization OF NOTCH (NICD) in PLK1-modulated melanoma cells. The data are presented as mean ± SEM with statistical significance compared to shNS or pcDNA control (*p < 0.05; ****p < 0.0001) in ≥2 biological replicates. For **a**, **c** and **d**, statistical significance was determined via multiple t-tests using the Holm–Sidak method. For **b**, statistical significance was determined using one- or two-way ANOVA followed by Fisher’s LSD tests.

We further asked if PLK1 has the ability to activate NOTCH in melanoma cells. To determine the effect of PLK1 modulation on NOTCH activation, we performed immunoblot analysis for NICD, the active form of NOTCH. The expression of NICD was significantly downregulated after PLK1 knockdown in A375, SK-MEL-2, and WM115 melanoma cells (Fig. 6a), and upregulated in PLK1 overexpressing and constitutively active PLK1 (T210D) containing A375 and WM115 cells. However, overexpression of wild-type PLK1 and its mutations (T210D, and K82R) in SK-MEL-2 cells did not change the NICD expression (Fig. 6b). We also performed immunofluorescence analysis for nuclear localization of NOTCH (NICD) and found that PLK1 knockdown melanoma cells showed significantly decreased NICD levels in the nucleus (Fig. 6c), while only PLK1 overexpressing A375 cells and constitutively active PLK1 (T210D) containing SK-MEL-2 melanoma cells showed increased levels of NICD in the nucleus (Fig. 6d). Immunofluorescence images are shown in Supplementary Fig. 5.

### PLK1 possesses a strong positive correlation with N-cadherin in human melanoma

**Fig. 7:**
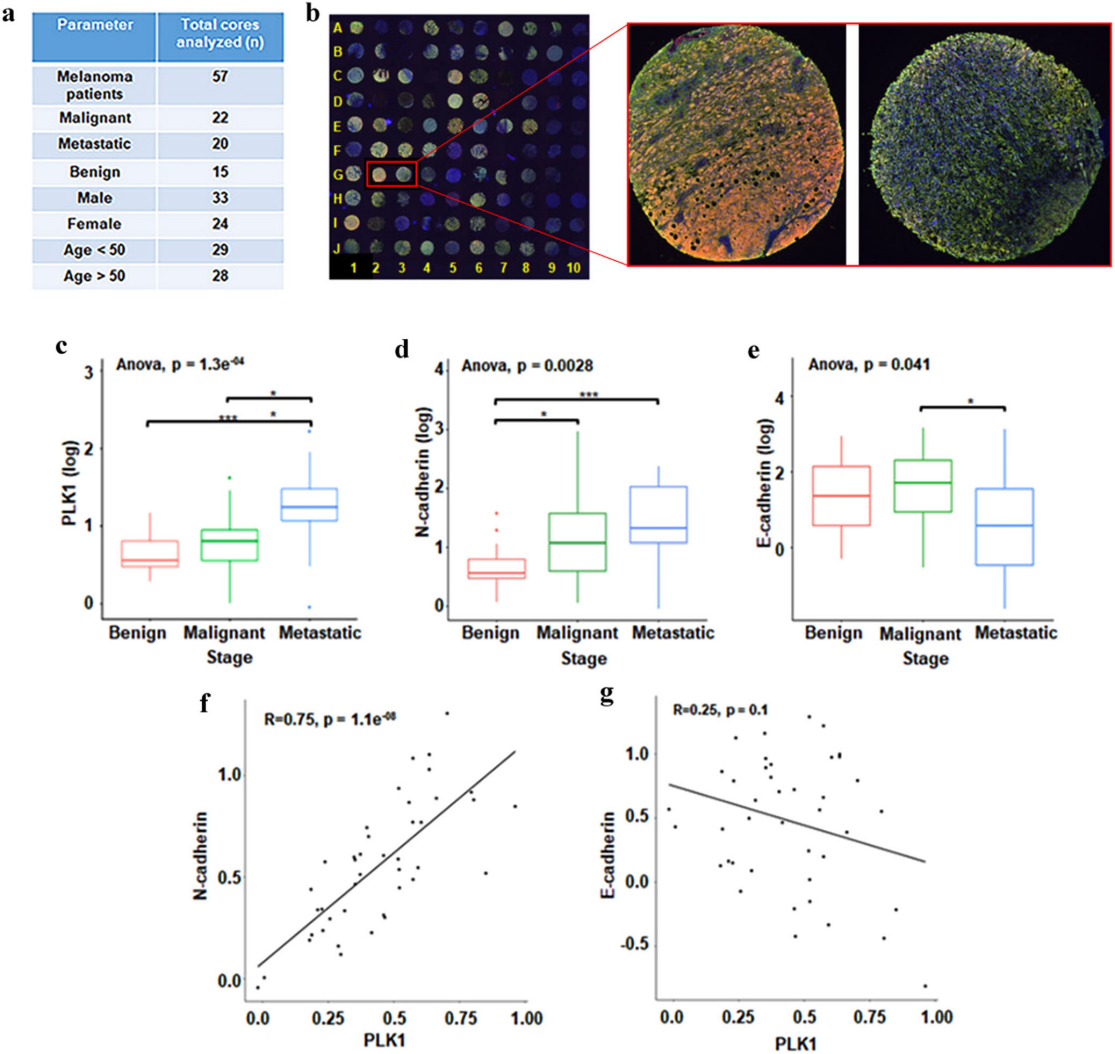
Human melanoma tissue microarray (TMA) analysis for PLK1, and EMT proteins N-cadherin and E-cadherin. **a** Summary of melanoma patients included in TMA analysis. **b** Mosaic image showing multiplex immunostaining of all melanoma tissue cores in the TMA with representative images of two selected melanoma tissue cores showing differential expression of PLK1 (Green), N-cadherin (Amber) and E-cadherin (Red). **c** Expression of PLK1, **d** Expression of N-cadherin, and **e** Expression of E-cadherin, in melanoma tissue cores. Statistical analyses were conducted using the R software (v3.5.1) and its developmental environment RStudio (v1.1.423). Statistical significance was obtained using Wilcoxon test (*p < 0.05; ***p < 0.001). **f** Correlation analysis between PLK1 and N-cadherin. **g** Correlation analysis between PLK1 and E-cadherin. For a simple linear regression study, the R package “ggpubr” (v0.1.8) was implemented to plot the correlation between PLK1 ~ N-cadherin, and PLK1 ~ E-cadherin.

To determine the clinical significance of our in vitro cell culture findings, we employed a commercially available human melanoma tissue microarray (TMA) coupled with high-throughput, multispectral Vectra scanning and inForm analysis, which allowed us to objectively analyze and quantify protein levels in 57 clinical tissue specimens of nevus, primary and metastatic melanoma (Fig. 7a). The TMA was co-immunostained for PLK1, N-cadherin, E-cadherin, and the melanoma biomarker S100. The multicolor stained mosaic TMA image is shown in Fig. 7b, including representative images of multiplex immunostained melanoma tissues showing differential expression of PLK1 (Green), N-cadherin (Amber), and E-cadherin (Red). Representative single-color images are shown in Supplementary Fig. 6. We observed an increase in PLK1 expression in primary and metastatic melanoma as compared to benign nevi, with a significant increase in metastatic tumors compared to primary tumors (Fig. 7c), which is in agreement with previously published data^5^. Similarly, N-cadherin expression was markedly higher in metastatic melanoma (fold-change=2.31) and primary melanoma (fold-change=1.8) when compared to benign nevi (Fig. 7d). Further, we observed a significant decrease in E-cadherin expression in metastatic tumors compared to primary melanoma (fold-change = −2.53) (Fig. 7e). Moreover, using single linear regression analyses between the expression of two proteins, we found a significant strong positive correlation between PLK1 and mesenchymal marker N-cadherin (correlation coefficient R = 0.75) (Fig. 7f). We also found a weak but negative correlation between PLK1 and epithelial marker E-cadherin with correlation coefficient R = −0.25 (Fig. 7g).

### PLK1, NUMB, and NOTCH affect the overall survival of patients with melanoma

**Fig. 8:**
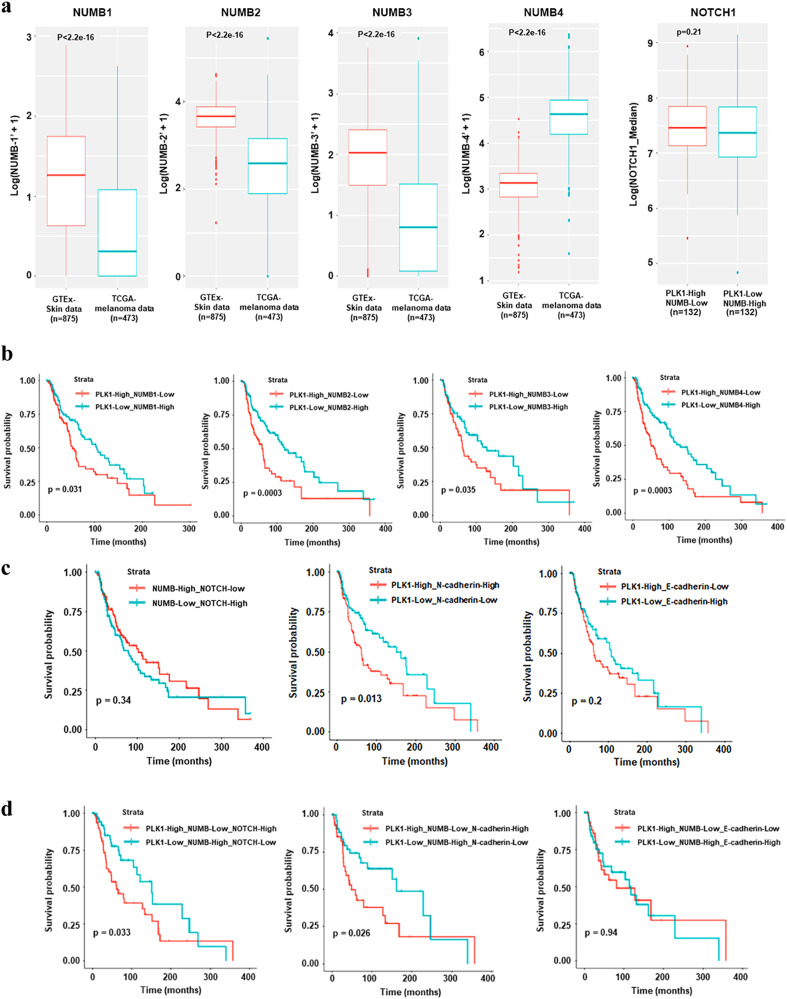
Analysis of TCGA melanoma and GTEx healthy individual databases for expression of NUMB isoforms and overall survival (OS) of melanoma patients. **a** Transcript level expression of four NUMB isoforms in melanoma patients compared to healthy individuals. **b** The OS was compared between melanoma patients with “high PLK1 and low NUMB-isoforms” (red, levels > median) and “low PLK1 and high NUMB-isoforms” (cyan, levels < median). **c** The OS was compared between melanoma patients with “high NOTCH1 and low NUMB” (red, levels > median) and “low NOTCH1 and high NUMB” (cyan, levels < median); “high PLK1 and high N-cadherin” (red, levels > median) and “low PLK1 and low N-cadherin” (cyan, levels < median); “high PLK1 and low E-cadherin” (red, levels > median) and “low PLK1 and high E-cadherin” (cyan, levels < median). **d** The OS was compared for combined high/low expression of triple genes PLK1, NUMB, and NOTCH; PLK1-NUMB-N-cadherin; and PLK1-NUMB-E-cadherin between melanoma patients. For OS analysis (n = 479 patients with melanoma from TCGA melanoma database), Kaplan-Meier curves were plotted using the R package “survminor” (v0.4.6). OS Overall survival.

The NUMB gene has been reported to be alternatively spliced to generate six functionally distinct isoforms, out of which four transcript variants (NUMB1-651 aa; NUMB2-603 aa; NUMB3-640 aa; NUMB4-592 aa), are most prevalent. We determined the expression of all four NUMB isoforms in melanoma by comparing TCGA data and GTEx data. Our data demonstrated that NUMB isoforms 1-3 were downregulated and isoform 4 was upregulated in melanoma (Fig. 8a). This was also validated on isoform expression data derived from the public cancer portal GEPIA2 (Gene Expression Profiling Interactive Analysis), which refers to TCGA and GTEx datasets^20^. Additionally, as NUMB is a known NOTCH inhibitor, we analyzed the expression of NOTCH1 using TCGA melanoma patient data and found a non-significant decreasing trend in NOTCH1 expression with PLK1-low/NUMB-high expression when compared to PLK1-high/NUMB-low levels (Fig. 8a).

**Fig. 9:**
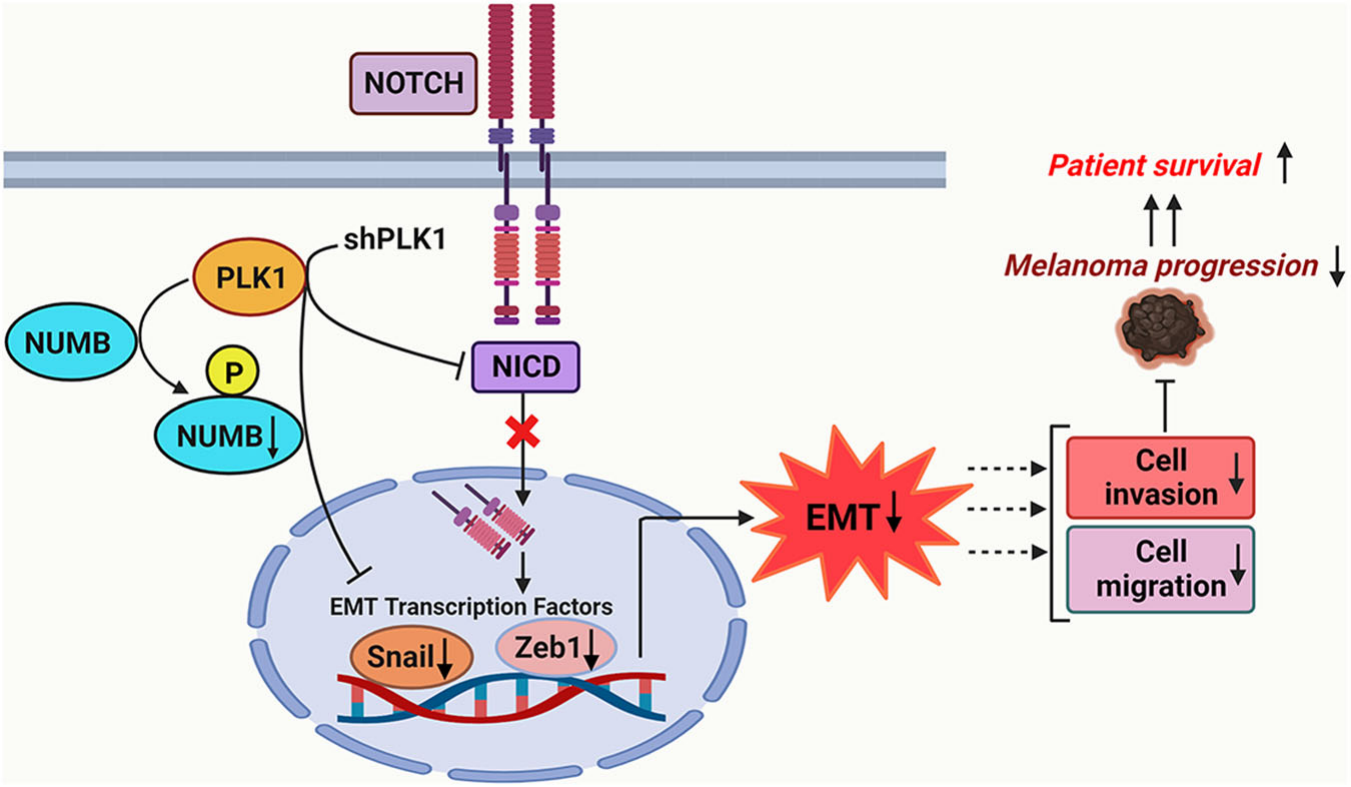
Proposed model of PLK1, NUMB, and NOTCH in melanoma progression. Based on our in vitro human melanoma cell models and ex vivo human melanoma TMA study as well as TCGA melanoma database analysis, we identified a previously unknown PLK1 phosphorylation site on NUMB protein that might be involved in melanoma cell migration, invasion, and EMT. Additionally, our study suggested that PLK1 inhibition altered the NOTCH pathway and EMT-related molecular consequences, which may ultimately lead to improved survival of patients with melanoma. This figure was created using BioRender.

To examine how the combined expressions of PLK1-NUMB, NOTCH-NUMB, PLK1-N-cadherin and PLK1-E-cadherin affect overall survival (OS) of melanoma patients, we downloaded the RNA-Seq gene expression and clinical data for a total of 479 patients from The Cancer Genome Atlas (TCGA) melanoma data set^21^. The Kaplan-Meier survival curve showed that the expression of PLK1 and NUMB (isoforms 1-4) were inversely associated with survival of melanoma patients as high PLK1 with low NUMB showed significant decrease in OS compared to low PLK1 and high NUMB (Fig. 8b). Further, high NOTCH with low NUMB expression was also associated with a non-significant trend of decreased OS (Fig. 8c). Interestingly, high PLK1 with high N-cadherin expression was associated with significantly shorter OS. Although not significant, there was a trend of improved OS with low PLK1 and high E-cadherin expression (Fig. 8c). When we determined the association of combined expression of these three genes in melanoma patient survival, we found that patients with ‘high PLK1 with low NUMB and high NOTCH’ and ‘high PLK1 with low NUMB and high N-cadherin’ have significantly decreased OS (Fig. 8d). Overall, these results suggest the significance of PLK1, NUMB, and NOTCH signaling in melanoma progression and patient survival (Fig. 9).

## Discussion

On the molecular level, EMT can be recognized by the reduced expression of epithelial markers such as E-cadherin and some cytokeratin isoforms, and the elevated expression of mesenchymal markers such as N-cadherin, and Vimentin^12^. Importantly, the loss of cell–cell contacts and the reorganization of the intracellular cytoskeleton during EMT result in increased cell migration and invasion^7^, which allows cells to invade the surrounding stroma and vasculature, thereby leading to tumor dissemination and metastases. Despite the non-epithelial nature of melanoma cells, similar EMT characteristics, including an E-cadherin/N-cadherin switch, have been shown to promote melanoma invasion and metastasis^22–24^. Thus, EMT-related proteins as well as EMT-modulating upstream signaling molecules have been considered as potential therapeutic targets against melanoma metastasis^25^. An early and key event in metastatic progression is the loss of cell–cell interactions, and N-cadherin has been reported to play a crucial and central role in this process. Further, N-cadherin knockdown using specific siRNA has been shown to inhibit the invasion ability of human melanoma cells^26^.

In this study, first, using Affymetrix microarray analysis, we demonstrate that shRNA-based PLK1 inhibition modulates EMT and metastasis-related genes and pathways in melanoma cells. Further, using in vitro experiments with PLK1 overexpression, knockdown, and kinase active (T210D)/inactive (K82R) modulations^27–29^ in multiple melanoma cell lines, we found that both PLK1 expression and kinase activity are important in promoting cell migration, invasion and EMT in melanoma. Importantly, we observed that shRNA-mediated PLK1 knockdown in A375, WM115, and SK-MEL-2 cells (human melanoma cell lines that possess high metastatic potential) inhibited cell migration and invasion, as well as modulated the expression of EMT-related proteins.

Previously, we have also demonstrated an association of PLK1 with NUMB^6^, although it is not fully known how the two proteins interact with each other during melanoma progression. PLK1 has been reported to directly phosphorylate NUMB at serine 265 and 284, though it was found using p65 NUMB isoform 4^30^. Since NUMB has also been linked with EMT regulation^9,17–19^, and the fact that PLK1 can directly interact with NUMB, we determined the putative PLK1 phosphorylation site(s) on NUMB. For this, we utilized purified recombinant proteins, PLK1 and NUMB (isoform 1, also known as transcript variant 1; 651 aa)^31^. Utilizing in vitro kinase assay followed by mass spectrometry, we identified Serine 413 (S413) as a putative PLK1 phosphorylation site of NUMB. This phosphorylation was detected by phospho-specific NUMB antibody (p-S413-NUMB) and further confirmed using site-directed mutagenesis where S413A-mutated NUMB protein did not show phosphorylation at S413 after in vitro kinase assay. Further, overexpression of WT NUMB, a non-phosphorylatable (S413A) and a phosphomimetic mutant (S413D) of NUMB in multiple melanoma lines showed involvement of NUMB S413 phosphorylation in cell migration, invasion, and EMT-related changes without affecting cell proliferation. Interestingly, ectopic expression of NUMB inhibited invasion, and the S413D phosphomimetic mutant rescued NUMB-inhibited invasion, which was consistent in all three melanoma cell lines tested. These results support the previously undefined and central mechanism in this study, showing the importance of phosphorylation of NUMB by PLK1. However, we found some inconsistent changes in EMT-related proteins in these three melanoma cell lines with WT NUMB and S413A/S413D mutations. The differences in expression for EMT-related protein could be due to the genetic drivers specific to each cell line (A375: BRAF V600E, SK-MEL-2: NRAS, WM115: BRAF V600D) that might have different downstream signaling effectors. Additionally, these results might be influenced by MITF (Microphthalmia-associated transcription factor) signaling, an important factor in melanoma EMT as A375 and SK-MEL-2 cells are MITF^low^ and WM115 cells has MITF^high^ phenotype^32,33^. Additionally, these results are in line with other published studies in which NUMB modulation did not affect the proliferation of melanoma cells^7^, and NUMB isoform 1 inhibited migration and invasion by preventing EMT in esophageal cancer^34^. However, these changes appear to be independent of NOTCH activation as we did not observe a significant change in NICD expression upon NUMB modulation in melanoma cells. The role of the NUMB protein in inhibiting NOTCH signaling may be dependent on the context of variety in NUMB isoforms. Interestingly, using TCGA and GTEx databases, we found that NUMB isoforms -1, -2, and -3 were downregulated, while isoform-4 was upregulated in melanoma. However, further studies are required to delineate the role and functional significance of different NUMB isoforms in melanoma progression.

In an earlier study, we found a positive correlation between PLK1 and NOTCH in human melanoma tissues^35^. In this study, we were able to demonstrate that shRNA-mediated inhibition of PLK1 in the melanoma cell lines (A375 and SK-MEL-2) inhibited the NOTCH pathway by decreasing the expression of NICD (NOTCH intracellular domain), as well as its nuclear intensity. However, the changes in NICD expression and nuclear intensity due to the overexpression of PLK1, and its mutant constructs K82R, and T210D were not consistent in all melanoma cell lines tested. As PLK1 is already upregulated during melanoma progression, further forced overexpression may not be able to produce significant differences to visualize at the molecular level.

NICD is known to translocate into the nucleus and functions as a transcriptional regulator that increases the expression of SNAIL. Further, SNAIL functions as a repressor of E-cadherin, which is a crucial step in reducing cell-cell adhesion, increasing cellular movement resulting in EMT acquisition^36^, and an increase in N-cadherin expression. Thus, the effects of PLK1 on EMT in melanoma could be mediated through the NOTCH pathway, which is shown to stimulate the EMT process in other cancers^37,38^. Based on our TMA analysis, we observed an increase in PLK1 expression in primary and metastatic melanoma as compared to benign nevi, with a significant increase in metastatic tumors compared to primary tumors. This is in accordance with our previously published study on PLK1 expression in melanoma patient tissues^39^. Similarly, N-cadherin expression was markedly higher in metastatic melanoma and primary melanoma when compared to benign nevi, which is in accordance with a previously published study showing increasing levels of N-cadherin during melanoma progression^40^. Further, we observed a significant decrease in E-cadherin expression in metastatic tumors compared to primary melanoma. These results correspond to an earlier published study analyzing the expression of E-cadherin and N-cadherin in primary and metastatic melanoma patient tissues^41^. Moreover, using single linear regression analyses between expressions of two proteins, we found a significant strong positive correlation between PLK1 and mesenchymal marker N-cadherin. We also found a weak but negative correlation between PLK1 and epithelial marker E-cadherin. These results suggest that the expression of PLK1 together with the expression of N-cadherin may be an important indicator of clinical melanoma prognosis. This is also supported by melanoma TCGA data analysis where we found that composite high mRNA expressions of PLK1 and N-cadherin significantly decreased overall survival of melanoma patients, and low PLK1 with high E-cadherin expression was associated with a trend of higher overall survival. Additionally, PLK1 and NUMB expressions were inversely associated with melanoma patient survival as high PLK1 with low NUMB (all isoforms 1-4) showed a significant decrease in patient survival compared to low PLK1 and high NUMB. These results are also in accordance with another published study in which the authors used IHC analyses and confirmed a positive correlation between N-cadherin and NOTCH1 expression in the same melanoma tumor samples and a concomitant high expression of N-cadherin and NOTCH1 was found to be associated with significantly low overall survival of melanoma patients^42^. Moreover, melanoma patient survival analysis taking into account all three genes showed that ‘high PLK1 with low NUMB and high NOTCH’ and ‘high PLK1 with low NUMB and high N-cadherin’ have significantly decreased overall survival. These observations suggest that melanoma tumors with PLK1-high/NUMB-low/NOTCH-high/N-cadherin-high/E-cadherin-low expression profile may have worse clinical outcomes associated with poor survival rates in patients with melanoma. Further, melanoma patients with high PLK1, and high NOTCH expression could be a candidate for combined targeting of PLK1 and NOTCH. Conversely, patients with low PLK1, high NUMB, and high NOTCH expression could benefit from the development of a potential modulator of NUMB phosphorylation.

In summary, our study provides mechanistic evidence using multiple human melanoma cell line models and clinical patient tissues that PLK1 is an important regulator of EMT, cell migration, and invasion, and thus, melanoma progression. In addition, the results obtained using mass spectrometry and site-directed mutagenesis suggest that PLK1 phosphorylates NUMB at Ser413, a phosphorylation site that was not known earlier. Our study also indicates that PLK1-mediated EMT changes may be interceded through phosphorylation of NUMB and/or NOTCH activation. As EMT has been considered a major determinant of melanoma metastasis, our study suggests a potential role of PLK1, NUMB, and NOTCH in the metastatic progression of melanoma that might be associated with poor survival of patients. Therefore, this study highlights the therapeutic significance of targeting PLK1, NUMB, and NOTCH in the clinical management of melanoma.

## Methods

### Cell lines and culture

Cell lines were obtained from ATCC. A375 and SK-MEL-2 cells were cultured and maintained in Dulbecco’s Modified Eagle’s Medium (DMEM), and WM115 cells in Minimum Essential Medium (MEM), with 10% FBS at standard cell culture conditions (37 °C, 5% CO_2_ in humidified incubator). All cell lines were routinely authenticated via STR testing at the University of Wisconsin Translational Research Initiatives in Pathology (TRIP) lab and tested routinely for mycoplasma using the Lonza Mycoalert Plus Kit.

### Cell line creation

A375, SK-MEL-2, and WM115 shNS and shPLK1 cell lines were created using lentivirally-transduced shNS and shPLK1 plasmids as described previously^6^. Stable polyclonal pools were created for use by selection with 2 µg/ml of puromycin. pcDNA plasmids containing WT, K82R, or T210D PLK1 sequences were a kind gift from Dr. Mark Burkard, UW-Madison. Empty vector (pCMV6-Entry #PS100001) and NUMB overexpression plasmids were purchased from Origene (WT NUMB #RC220960 with custom mutated plasmids containing S413A and S413D mutations). For creation of the PLK1 overexpression cell lines, these plasmids were transfected into A375, SK-MEL-2 and WM115 human melanoma cells using Lipofectamine 2000 per manufacturer’s recommendations, and selected using G418 antibiotic-containing media (2, 2.5, and 0.05 mg/ml, respectively). Cells were used as a polyclonal pool for further experiments and grown in selection media.

### RNA extraction and GeneChip human transcriptome array 2.0

Previously created^4^ A375 shNS and shPLK1 cells were plated in a 6-well plate (1×10^4^ cells per well) and treated with doxycycline to induce expression. After 48 h, cells were collected and RNA isolated using RNeasy Plus Mini Kit (QIAGEN, CA). Three replicates of each cell line were made. The concentration and quality of RNA were checked by NanoDrop 2000 (ThermoFisher Scientific) and Agilent Bioanalyzer (Agilent Technologies, Santa Clara, CA). The RNA samples were hybridized to the Affymetrix Human Transcriptome Array (HTA) 2.0 microarrays and processed by the UW-Madison Gene Expression Center in the UW Biotechnology Center for Affymetrix GeneChip Services.

### Microarray data analysis

The raw data of the Affymetrix gene chip were imported into the Affymetrix Transcriptome Analysis Console v.3.0.0.466 (TAC) and processed using the robust multi-array (RMA) method followed by analysis using Affymetrix Expression Console software. The differentially expressed genes (DEG) between shRNA-NS (non-sense control) and shRNA-PLK1 were identified based on whether the p-value was smaller than 0.01. The DEGs were then submitted for Ingenuity Pathway Analysis (IPA; Qiagen Bioinformatics) to identify canonical pathways significantly altered by PLK1 knockdown. *p* < 0.05 was used as a threshold for significant pathways.

### Cell proliferation assay (RealTime-Glo MT)

RealTime-Glo MT Cell Viability Assay (Promega) was performed per the manufacturer’s protocol. Briefly, 1×10^3^ cells were plated in quadruplicate in white clear-bottom 96-well plates with RealTime-Glo reagents. Luminescence readings were taken at the indicated time points using a BioTek Synergy H1 Multimode plate reader with Gen5 software (v. 3.11) and normalized to the initial (1 h) reading.

### Scratch assay for migration of cells

PLK1-knockdown cells or control A375 cells (1×10^5^ cells) were seeded into 6-well plates and incubated at 37 °C and 5% CO_2_. Once cells reached to confluency (24-48 h), a wound was created by scratching the confluent monolayer with a 20 µl pipette tip. Cell migration was assessed at various time points and the images of cells were acquired at 0, 24, and 48 h post-wounding using an EVOS core XL microscope at 20x magnification. Quantification of wound area was performed using an established ImageJ automated method^43^.

### Matrigel invasion assay

Cell invasion assay was performed using Matrigel Invasion Chambers (BD Bioscience) per the manufacturer’s protocol. Briefly, 2.5×10^4^ cells in medium (without FBS) were seeded in matrigel-coated transwells in 24-well plates. Chemoattractant (media with 10% FBS) was added to the lower chamber of the plate which was further incubated at 37 °C and 5% CO_2_. After 24 h cells were stained with 1% crystal violet for 1 h. The cells that migrated to the bottom of Matrigel membrane were imaged using an EVOS XL Core microscope at 20X magnification. Quantification of cell numbers was performed using ImageJ automated method^43^.

### Protein isolation and immunoblot analysis

Proteins were isolated from melanoma cell pellets using 1x RIPA buffer (Millipore #20-188) supplemented with Protease Inhibitor Cocktail (ThermoFisher #87786) and PMSF (Amresco #0754) and quantified using BCA Assay (ThermoFisher). Primary antibodies against PLK1 (#4513, 1:20), NUMB (#2756, 1:40), NOTCH1 (NICD) (#3608, 1:50), E-cadherin (#3195, 1:20), N-cadherin (#13116, 1:50X), Vimentin (#5741, 1:50), ZEB1 (#3396, 1:100), and SNAIL (#3879, 1:50) were purchased from Cell Signaling Technologies (CST). Immunoblotting was performed by capillary-based Simple Western method using the ProteinSimple Jess system, employing a 12-230 KDa Separation Module and anti-rabbit secondary antibody per manufacturer instructions and as previously described^44^. The primary antibody against pS413-NUMB was purchased from GenScript as a custom antibody development order and was used in traditional western blotting to analyze NUMB phosphorylation at S413 after in-vitro kinase assay with PLK1. Uncropped immunoblots and total protein assay images for Simple Western analysis are included in Supplementary Figs. 7-11.

### Immunofluorescence analysis

Melanoma cells (5×10^4^ cells/chamber) were seeded and grown in 8-well chamber slides (Nunc, Thermo Scientific) in culture medium and allowed to adhere overnight. Immunofluorescence staining of NOTCH1 (ab52627 Abcam; 1:150), N-cadherin (13116 CST; 1:400) and E-cadherin (3195 CST; 1:400) was performed. Briefly, cells were fixed with 4% paraformaldehyde and permeabilized with 0.5% Triton X-100 in PBS and then blocked for 1 h at room temperature in 2% BSA/0.1% Triton X-100 in PBS. Cells were stained with primary antibodies overnight at 4 °C, washed, and incubated with Alexa Fluor 488 or 594 secondary antibodies (ThermoFisher Scientific; 1:500-1:1000) for 1 h at room temperature. Counterstaining was performed using 4’,6-diamidino-2-phenylindole (DAPI)-containing Prolong Diamond Mounting media (ThermoFisher Scientific). The E- and N-cadherin images were visualized using EVOS FL Auto Imaging System (ThermoFisher Scientific) and quantification was performed using Celleste 6 Image Analysis Software (ThermoFisher Scientific). The NOTCH1 images were visualized, and nuclear notch protein was quantified using the BioTek Lionheart FL microscope and Gen5 software (v.3.11). For quantification, 20x images were taken of each line and Cellular Analysis was performed using the settings in Supplementary Table 2.

### In vitro kinase assay

Purified PLK1 (Sigma #P0060-10UG), NUMB (Origene #TP320960) and mutated NUMB (S413A and S413D: Origene #TP605384 and #TP607760, respectively) proteins were obtained commercially. In vitro kinase assay was performed as described earlier^45^. PLK1 (20 ng) and NUMB (1 µg) proteins were incubated in kinase buffer (20 mM HEPES, pH 7.4, 150 mM KCl, 10 nM MgCl2, 1 mM EGTA, 0.5 mM dithiothreitol, 5 mM NaF) in presence of 100 mM ATP for 30 min at 30 °C. Phosphorylation reactions were stopped by adding SDS sample buffer (Bio-Rad #1610747, which were then heated for 5 min at 95 °C before analysis by immunoblot analysis with specific antibodies.

### Mass spectrometry analysis to identify putative phosphorylation site

After in vitro phosphorylation, the corresponding NUMB protein band on the SDS gel stained with coomassie brilliant blue was excised out for in-solution digestion with trypsin followed by bottom-up mass spectrometry. After MS/MS analysis of the resulting peptides, the data were examined with Mascot v2.4 (Matrix Science, London) to identify the amino acid misincorporations using an error-tolerant search, which is one of the optional modes of the Mascot protein database search^46^. In this analysis, the raw data are initially searched against a reference protein database, after which the MS/MS data that do not match the expected amino acid sequences of known proteins are checked against a database containing all possible amino acid misincorporations and posttranslational modifications. The peptides containing phosphorylated Serine residues can be identified by a loss of phosphoric acid (98 Da)^47^.

### Clustal omega multiple sequence alignment of NUMB protein

NUMB protein sequences from various species were obtained from SwissProt database^48^. Multiple sequence alignment was performed using protein sequences of NUMB and standard Clustal Omega analysis^49^, which allows to identify the conserved amino acid residues represented by an asterisk (*) and similarly charged amino acid residues shown by a colon (:).

### Tissue microarray (TMA) and multicolor immunofluorescent staining

Human melanoma TMA containing samples collected under HIPAA-compliant protocols was purchased from US Biomax (Cat #ME1004f) and was stained as described previously by the UW Skin Disease Resource Center’s Experimental Cutaneous Pathology Core^39^. Briefly, TMA slide was deparaffinized in xylene, and rehydrated through a standard graded ethanol series. Antigen retrieval was performed in citrate buffer using microwave treatment (MWT). TSA visualization was performed with the Opal seven-color IHC Kit (NEL797B001KT, PerkinElmer) for DAPI, PLK1 (Abcam #17056, Opal 520), S100 (Ventana 4C4.9, Opal 540), E-Cadherin (CST #3195, Opal 570), and N-Cadherin (CST #13116, Opal 620). All multiplex TSA experiments were performed by repeating staining cycles in series, with MWTs in between each cycle and at the end of the multiplex TSA. Finally, all multiplex TSA staining was finished with a DAPI counterstain. TSA single-stained slides were finished with MWT.

### TMA image acquisition and analysis using InForm software

For quantitative staining analysis, Vectra 2.0 automated quantitative tissue imaging system with Nuance and inForm software 2.2.1 (PerkinElmer) was used as described previously^39^. The multiplexed TMA was imaged with the Vectra slide scanner, using a scanning protocol that was created based on core size and layout, as well as an acquisition of spectral library. For each slide, an 8-bit image cube from each of the TMA tissue cores was acquired and the inForm advanced image analysis software was used to segment tissues (melanoma versus others) to analyze the protein levels. Then, the target signals were quantitated after unmixing the spectral curves with InForm software. Continuous signal intensity (mean optical density per pixel) was generated for each identified tissue/cell type. Data were exported for further analysis. Quantitation for PLK1, N-cadherin, and E-cadherin was performed in S100-positive cells in melanoma tissue.

### Correlation analysis

All statistical analyses were conducted using the R software (v3.5.1) and its developmental environment RStudio (v1.1.423). In order to satisfy the normal assumption of linear correlation, the expression values of PLK1, N-cadherin, and E-cadherin were first transformed to log scale. For simple linear regression study, the R package “ggpubr” (v0.1.8) was implemented to plot the correlation between PLK1 ~ N-cadherin, and PLK1 ~ E-cadherin.

### TCGA data analysis

Clinical and RNA‑sequencing data from 479 patients with melanoma were downloaded from The Cancer Genome Atlas (TCGA) database using the cBioPortal for Cancer Genomics. To check how the combined gene expression affects the overall survival, the expression profile PLK1, NUMB, NOTCH, N-cadherin and E-cadherin and overall survival profile were first filtered to exclude censoring data. To visualize the survival plots, melanoma patients were separated into groups using the median value of gene expression as cutoff. For OS and DFS analysis (n = 479 patients with melanoma), Kaplan-Meier curves were obtained using the R package “survminor” (v0.4.6). The statistical differences between survival curves were assessed using log-rank tests.

### NUMB isoforms expression analysis using GTEx vs TCGA

Transcript data (expression of gene isoforms) for melanoma patients available from TCGA database were downloaded from https://gdac.broadinstitute.org/ and converted to TPM scale before analysis. GTEx skin isoform expression (v8.0) was extracted in TPM scale from www.gtexportal.org. NUMB isoforms from TCGA and GTEx databases were annotated by UCSC Genome Brower. In GTEx database, the NUMB isoform expressions were averaged for individuals with more than one skin sample isolated. The expression of NUMB isoforms between TCGA and GTEx databases were plotted as log2(TPM + 1) using the ggplot2 package (v3.4.0).

### Statistical analysis

Statistical analyses were performed using GraphPad PRISM 9.0 software (GraphPad Software, Inc., La Jolla, CA). Statistical significance was determined via multiple t-tests using the Holm–Sidak method for two experimental groups, and one- or two-way analysis of variance (ANOVA) followed by Fisher’s LSD tests for more than two experimental groups. Data are shown as mean ± SE unless otherwise specified. For TMA studies, statistical analysis was done in R software using ggboxplot and ggscatter.

### Supplementary information

Supplementary Material

## Data Availability

The microarray datasets generated in this article are available through Gene Expression Omnibus database (NCBI) (Accession Number GSE237385). Other experimental data supporting the findings of this study are available within the article and/or in the supplementary information. Materials generated in this study are available from the corresponding authors upon reasonable request. The gene expression and patient survival for TCGA melanoma cohort were accessed and downloaded from https://www.cbioportal.org/study/summary?id=skcm_tcga. The isoform expression for TCGA melanoma cohort was accessed and downloaded from http://firebrowse.org/?cohort=SKCM.

## Code availability

The scripts and code used for analyzing TCGA and GTEx data are available on github:

https://github.com/AhmadLab-UW/PLK1-NUMB-NOTCH-in-melanoma.

## References

[1] Switzer B, Puzanov I, Skitzki JJ, Hamad L, Ernstoff MS (2022). Managing Metastatic Melanoma in 2022: A Clinical Review. JCO Oncol. Pr..

[2] Cholewa BD, Liu X, Ahmad N (2013). The role of polo-like kinase 1 in carcinogenesis: cause or consequence?. Cancer Res..

[3] Gutteridge RE, Ndiaye MA, Liu X, Ahmad N (2016). Plk1 Inhibitors in Cancer Therapy: From Laboratory to Clinics. Mol. Cancer Ther..

[4] Gutteridge RE, Singh CK, Ndiaye MA, Ahmad N (2017). Targeted knockdown of polo-like kinase 1 alters metabolic regulation in melanoma. Cancer Lett..

[5] Schmit TL, Zhong W, Setaluri V, Spiegelman VS, Ahmad N (2009). Targeted depletion of Polo-like kinase (Plk) 1 through lentiviral shRNA or a small-molecule inhibitor causes mitotic catastrophe and induction of apoptosis in human melanoma cells. J. Invest. Dermatol..

[6] Schmit TL (2012). Numb regulates stability and localization of the mitotic kinase PLK1 and is required for transit through mitosis. Cancer Res..

[7] Hristova DM (2022). NUMB as a Therapeutic Target for Melanoma. J. Invest. Dermatol..

[8] Choi HY, Seok J, Kang GH, Lim KM, Cho SG (2021). The role of NUMB/NUMB isoforms in cancer stem cells. BMB Rep..

[9] Sheng W (2022). Numb-PRRL promotes TGF-beta1- and EGF-induced epithelial-to-mesenchymal transition in pancreatic cancer. Cell Death Dis..

[10] Lu Y (2015). Alternative splicing of the cell fate determinant Numb in hepatocellular carcinoma. Hepatology.

[11] Abballe L (2018). Numb Isoforms Deregulation in Medulloblastoma and Role of p66 Isoform in Cancer and Neural Stem Cells. Front. Pediatr..

[12] Ye X, Weinberg RA, Epithelial-Mesenchymal (2015). Plasticity: A Central Regulator of Cancer Progression. Trends Cell Biol..

[13] Krakhmal NV, Zavyalova MV, Denisov EV, Vtorushin SV, Perelmuter VM (2015). Cancer Invasion: Patterns and Mechanisms. Acta Nat..

[14] Wu J, Ivanov AI, Fisher PB, Fu Z (2016). Polo-like kinase 1 induces epithelial-to-mesenchymal transition and promotes epithelial cell motility by activating CRAF/ERK signaling. Elife.

[15] Cai XP (2016). PLK1 promotes epithelial-mesenchymal transition and metastasis of gastric carcinoma cells. Am. J. Transl. Res..

[16] Song R (2018). Effects of PLK1 on proliferation, invasion and metastasis of gastric cancer cells through epithelial-mesenchymal transition. Oncol. Lett..

[17] Cheng C (2020). Numb negatively regulates the epithelial-to-mesenchymal transition in colorectal cancer through the Wnt signaling pathway. Am. J. Physiol. Gastrointest. Liver Physiol..

[18] Liang J, Han B, Zhang Y, Yue Q (2019). Numb inhibits cell proliferation, invasion, and epithelial-mesenchymal transition through PAK1/beta-catenin signaling pathway in ovarian cancer. Onco Targets Ther..

[19] Hu XB, Ouyang LZ, He Y, Xia MZ (2019). Numb confers to inhibit epithelial mesenchymal transition via beta-catenin/Lin28 signaling pathway in breast cancer. Exp. Mol. Pathol..

[20] Tang Z, Kang B, Li C, Chen T, Zhang Z (2019). GEPIA2: an enhanced web server for large-scale expression profiling and interactive analysis. Nucleic Acids Res..

[21] Cancer Genome Atlas Network. (2015). Genomic Classification of Cutaneous Melanoma. Cell.

[22] Alonso SR (2007). A high-throughput study in melanoma identifies epithelial-mesenchymal transition as a major determinant of metastasis. Cancer Res..

[23] Caramel J (2013). A switch in the expression of embryonic EMT-inducers drives the development of malignant melanoma. Cancer Cell.

[24] Li FZ, Dhillon AS, Anderson RL, McArthur G, Ferrao PT (2015). Phenotype switching in melanoma: implications for progression and therapy. Front Oncol..

[25] Pearlman RL, Montes de Oca MK, Pal HC, Afaq F (2017). Potential therapeutic targets of epithelial-mesenchymal transition in melanoma. Cancer Lett..

[26] Ciolczyk-Wierzbicka D, Laidler P (2018). The inhibition of invasion of human melanoma cells through N-cadherin knock-down. Med. Oncol..

[27] Chun G (2010). Polo-like kinase 1 enhances survival and mutagenesis after genotoxic stress in normal cells through cell cycle checkpoint bypass. Carcinogenesis.

[28] Xiao D (2016). Polo-like Kinase-1 Regulates Myc Stabilization and Activates a Feedforward Circuit Promoting Tumor Cell Survival. Mol. Cell.

[29] Casenghi M, Barr FA, Nigg EA (2005). Phosphorylation of Nlp by Plk1 negatively regulates its dynein-dynactin-dependent targeting to the centrosome. J. Cell Sci..

[30] Shao C (2018). Plk1 phosphorylation of Numb leads to impaired DNA damage response. Oncogene.

[31] Santolini E (2000). Numb is an endocytic protein. J. Cell Biol..

[32] Vlckova K, Vachtenheim J, Reda J, Horak P, Ondrusova L (2018). Inducibly decreased MITF levels do not affect proliferation and phenotype switching but reduce differentiation of melanoma cells. J. Cell Mol. Med.

[33] Wardwell-Ozgo J (2014). HOXA1 drives melanoma tumor growth and metastasis and elicits an invasion gene expression signature that prognosticates clinical outcome. Oncogene.

[34] Hong J (2014). The tumor suppressive role of NUMB isoform 1 in esophageal squamous cell carcinoma. Oncotarget.

[35] Su S (2021). PLK1 and NOTCH Positively Correlate in Melanoma and Their Combined Inhibition Results in Synergistic Modulations of Key Melanoma Pathways. Mol. Cancer Ther..

[36] Wang Z, Li Y, Kong D, Sarkar FH (2010). The role of Notch signaling pathway in epithelial-mesenchymal transition (EMT) during development and tumor aggressiveness. Curr. Drug Targets.

[37] Zhang J (2018). Notch signalling induces epithelial‑mesenchymal transition to promote metastasis in oral squamous cell carcinoma. Int. J. Mol. Med..

[38] Li Y (2013). Regulation of EMT by Notch signaling pathway in tumor progression. Curr. Cancer Drug Targets.

[39] Cholewa BD, Ndiaye MA, Huang W, Liu X, Ahmad N (2017). Small molecule inhibition of polo-like kinase 1 by volasertib (BI 6727) causes significant melanoma growth delay and regression in vivo. Cancer Lett..

[40] Watson-Hurst K, Becker D (2006). The role of N-cadherin, MCAM and beta3 integrin in melanoma progression, proliferation, migration and invasion. Cancer Biol. Ther..

[41] Yan S (2016). Epithelial-Mesenchymal Expression Phenotype of Primary Melanoma and Matched Metastases and Relationship with Overall Survival. Anticancer Res..

[42] Murtas D (2017). Role of epithelial-mesenchymal transition involved molecules in the progression of cutaneous melanoma. Histochem. Cell Biol..

[43] Venter C, Niesler CU (2019). Rapid quantification of cellular proliferation and migration using ImageJ. Biotechniques.

[44] Chhabra G (2022). Antimelanoma Effects of Concomitant Inhibition of SIRT1 and SIRT3 in Braf(V600E)/Pten(NULL) Mice. J. Invest. Dermatol..

[45] Burkard ME (2009). Plk1 self-organization and priming phosphorylation of HsCYK-4 at the spindle midzone regulate the onset of division in human cells. PLoS Biol..

[46] Creasy DM, Cottrell JS (2002). Error tolerant searching of uninterpreted tandem mass spectrometry data. Proteomics.

[47] Tabb DL, Friedman DB, Ham AJ (2006). Verification of automated peptide identifications from proteomic tandem mass spectra. Nat. Protoc..

[48] Bairoch A, Apweiler R (2000). The SWISS-PROT protein sequence database and its supplement TrEMBL in 2000. Nucleic Acids Res..

[49] Sievers F (2011). Fast, scalable generation of high-quality protein multiple sequence alignments using Clustal Omega. Mol. Syst. Biol..

