# Supplementary material for: Role of PLK1/NUMB/NOTCH in epithelial-mesenchymal transition in human melanoma
Source: NPJ Precis Oncol. 2024 Jan 6;8:6. doi: 10.1038/s41698-023-00493-7 (PMC10771520; doi:10.1038/s41698-023-00493-7)
Supplement: Supplementary file 1 — Supplementary Material [file 41698_2023_493_MOESM1_ESM.pdf]

Supplementary Material

Role of PLK1/NUMB/NOTCH in epithelial-mesenchymal transition in human melanoma

Gagan Chhabra[1], Chandra K. Singh[1], Mary A. Ndiaye[1], Shengqin Su[1], Carl A. Shirley[1], Nihal Ahmad[1,2*]

Affiliation of authors:

[1] Department of Dermatology, University of Wisconsin, Madison, Wisconsin, 53706, USA;

[2] William S. Middleton VA Medical Center, Madison, Wisconsin, 53705, USA

[*]Correspondence to:

Nihal Ahmad, Ph.D., Department of Dermatology, University of Wisconsin,

1111 Highland Avenue, Room 7045, Madison, Wisconsin, 53705

Phone: (608) 263-2532; Fax: (608) 263-5223; E-mail: nahmad@dermatology.wisc.edu

Supplementary Figure 1. IPA analysis of Affymetrix Human Transcriptome Array (HTA) 2.0 microarray data for cumulative actions of altered genes due to PLK1-knockdown.

a

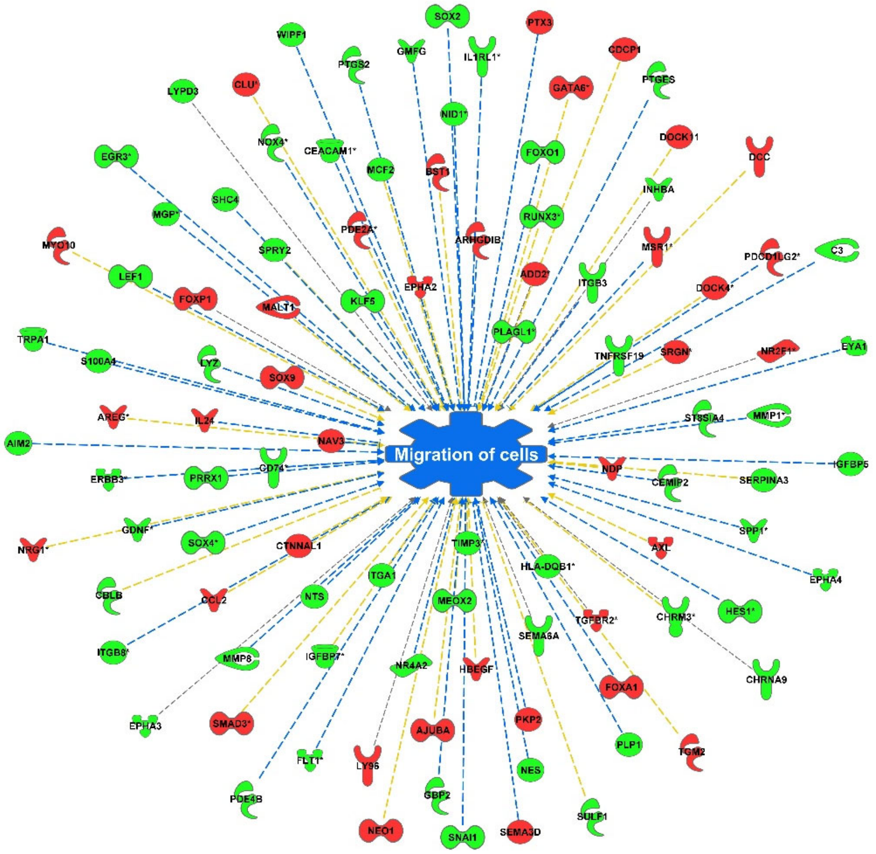

b

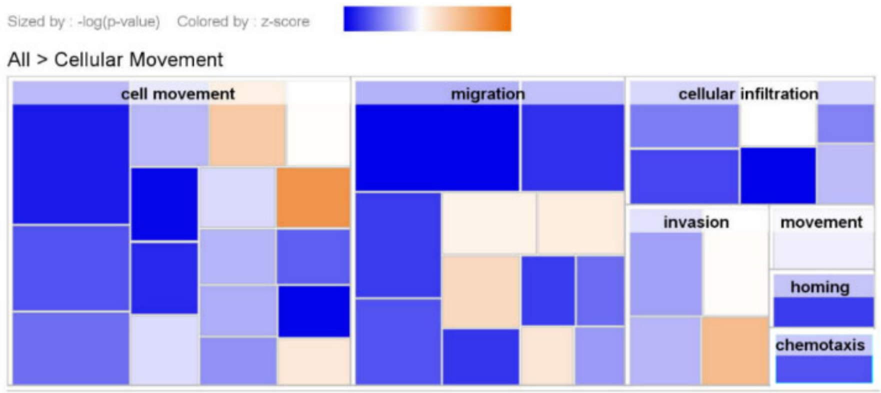

a IPA analysis predicted the inhibition of cell migration. Green and red icons correspond to significantly downregulated or upregulated genes, respectively. Blue represents 'inhibition' and gray and yellow lines represent 'unpredicted' or 'inconsistent' findings, respectively. **b** Heatmap representing diseases and functions affected in response to PLK1 knockdown in A375 melanoma cells. This heatmap is based on Z-scores where higher is represented by orange means activation, whilst lower by blue means inhibition. Each rectangle represents a functional category. Their size reflects the P values significance, i.e. the most significantly enriched categories with the largest rectangles. Overall, heatmap data predict inhibition (indicated by blue color) of most of the cancer-related processes and functions in response to PLK1 knockdown.

Supplementary Figure 2. Immunofluorescence analysis of E-cadherin and N-cadherin after PLK1 knockdown in SK-MEL-2 melanoma cells.

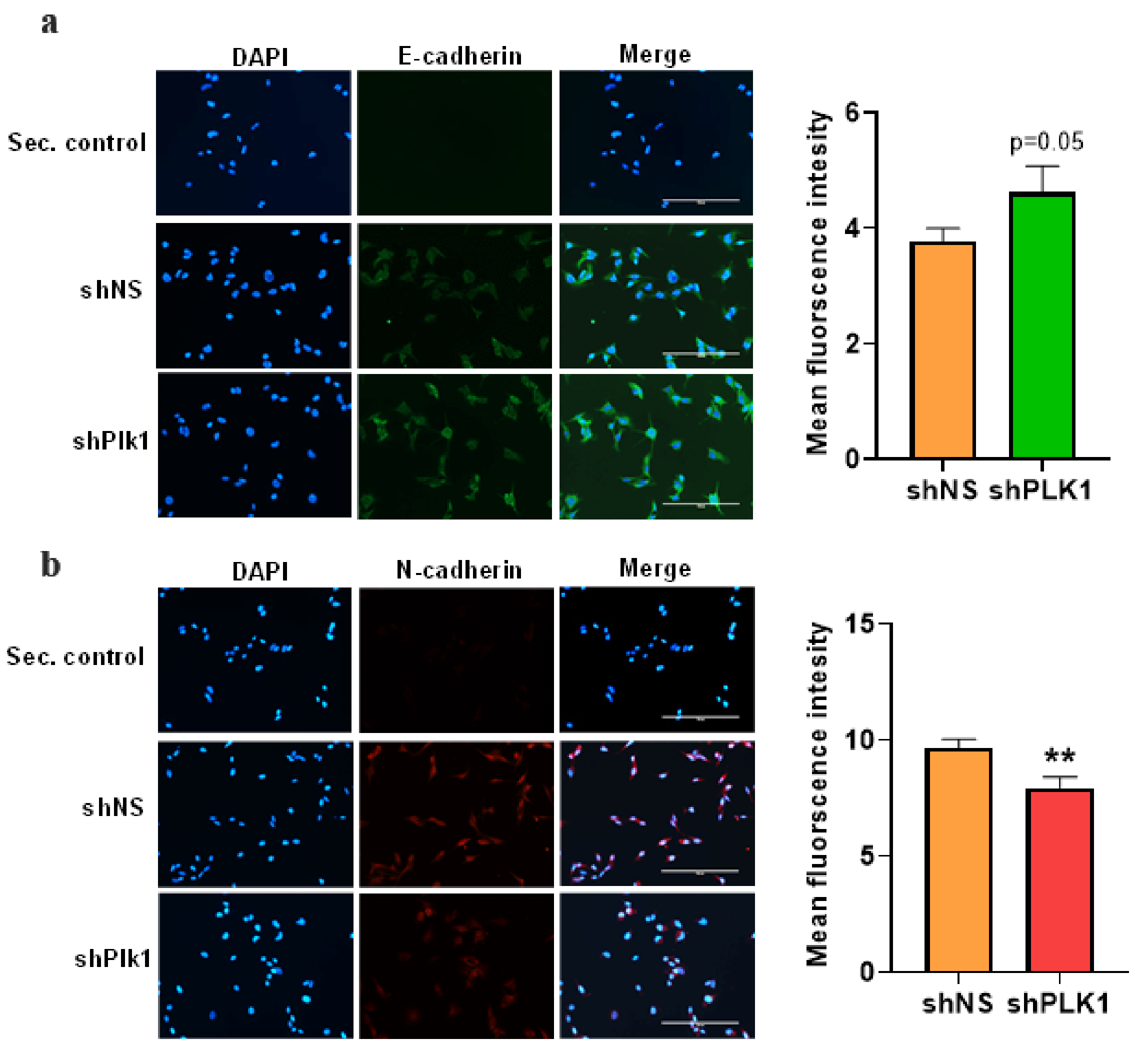

Immunofluorescence analysis was performed for **(A)** E-cadherin, and **(B)** N-cadherin in SK-MEL-2 melanoma cells. Images were visualized using EVOS FL Auto Imaging System (ThermoFisher Scientific) and quantification was performed using Celleste 6 Image Analysis Software (ThermoFisher Scientific) after normalization with background staining. Scale bar- 200 µm. The data are presented as mean ± SEM with statistical significance compared to shNS control (**p<0.01).

Supplementary Figure 3. Confirmation of PLK1 phosphorylation site (S413) on NUMB after *in vitro* kinase assay.

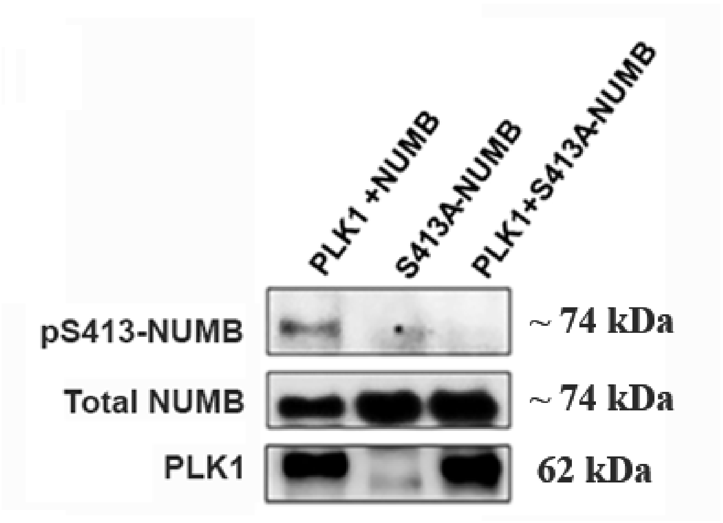

Immunoblot analysis for p-S413-NUMB, total NUMB, and PLK1 after *in vitro* kinase assay using PLK1 and S413A mutated NUMB protein by site-directed mutagenesis. All blots were derived from the same experiment and they were processed in parallel.

Supplementary Figure 4. Expression of PLK1, NICD, and EMT-related proteins in NUMB-modulated melanoma cells.

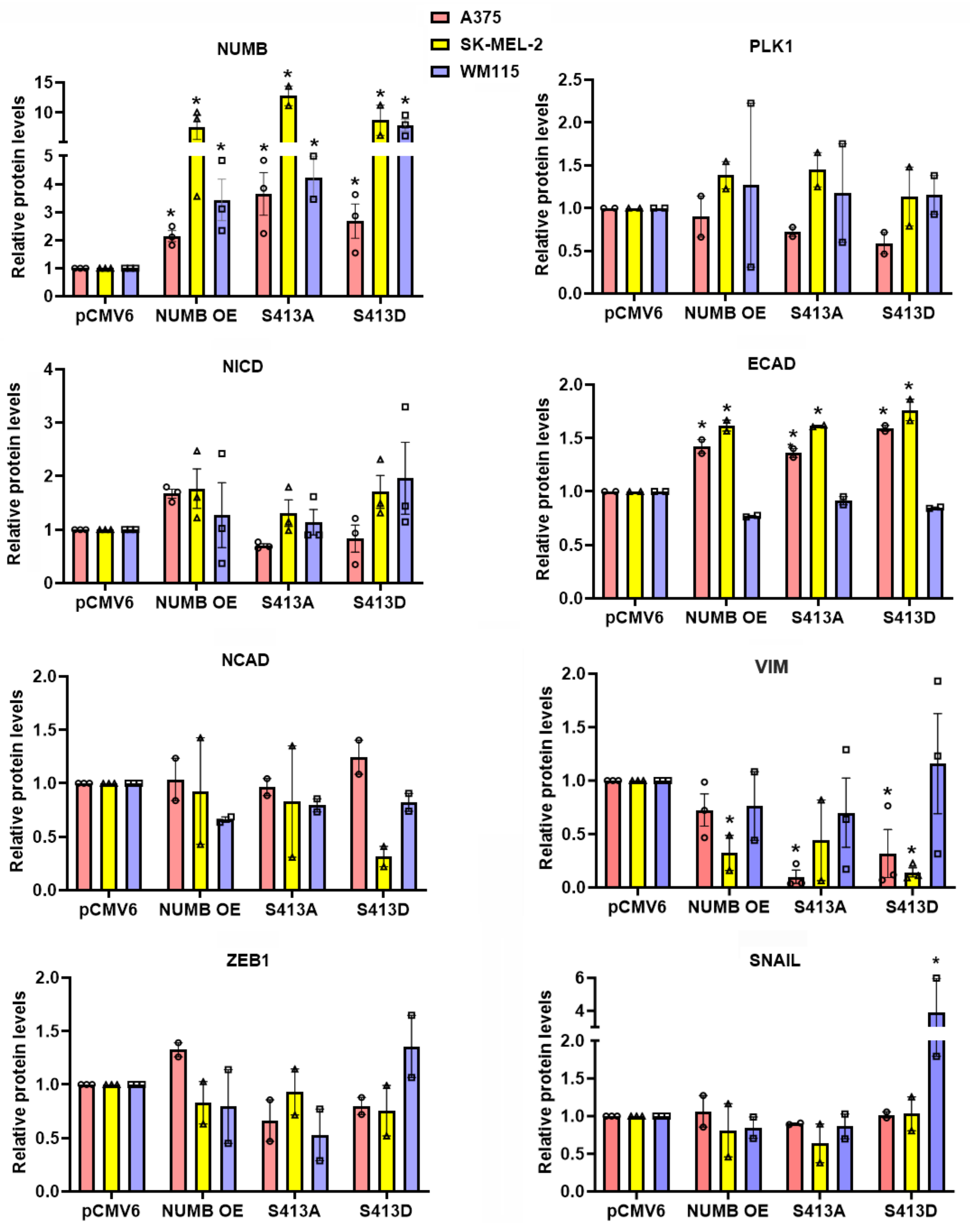

Relative PLK1, NUMB, NICD, and EMT-related protein levels were analyzed by Simple Western immunoblotting in NUMB-modulated melanoma cells. Data are presented as mean ± SEM with significance compared to pCMV6 control (*$p<0.05$) in at least 2 biological replicates. Statistical significance was determined using one- or two-way ANOVA followed by Fisher's LSD tests.

Supplementary Figure 5. Immunofluorescence analysis for Notch1 nuclear localization after PLK1 modulation in melanoma cells.

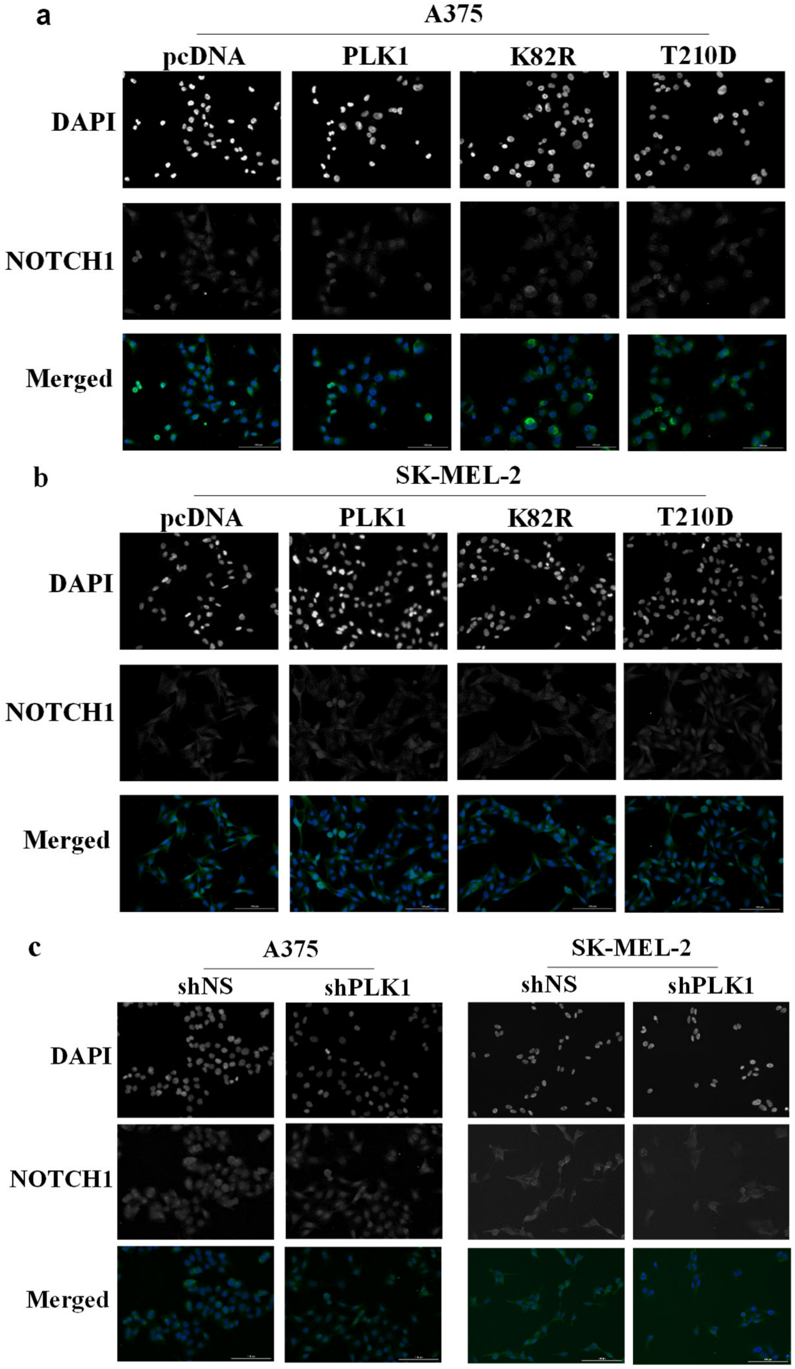

Representative immunofluorescence images for NOTCH1 nuclear localization using empty vector (pcDNA), PLK1 overexpressing, constitutively kinase-active (T210D), and kinase-inactive (K82R) in **a** A375, **b** SK-MEL-2, and **c** PLK1 knockdown (shPLK1), and non-sense (shNS) A375 and SK-MEL-2 cells. Scale bar- 100 µm.

Supplementary Figure 6. Representative single-color tissue microarray (TMA) images.

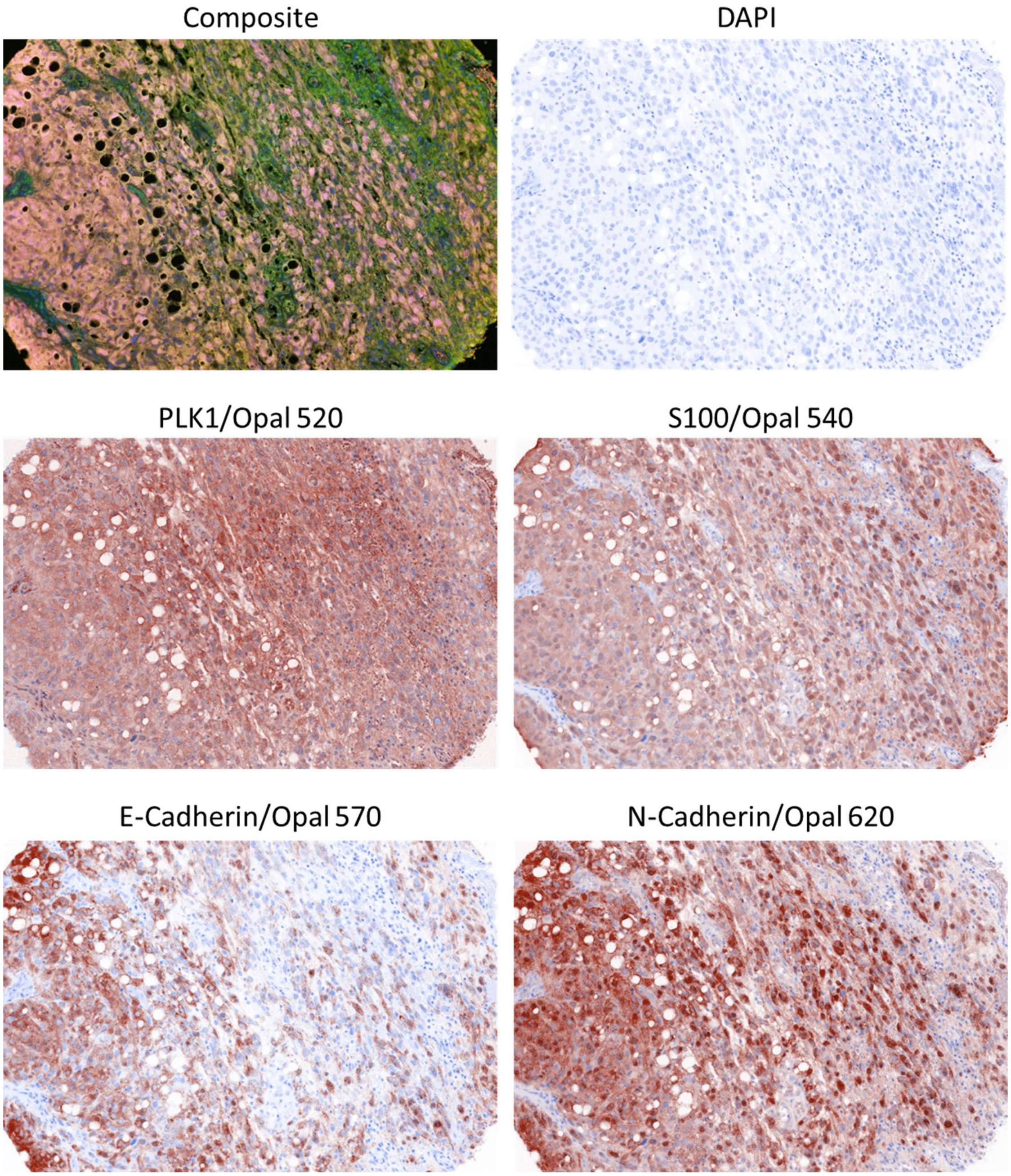

Representative images of TMA staining. The Vectra system provides a composite pseudocolored image along with DAB-mimicking pathology view images for each individual fluorophore. Protein staining is indicated above each image.

Supplementary Figure 7. Uncropped Simple Western immunoblots and total protein assay images for immunoblot data shown in Figure 2.

PLK1

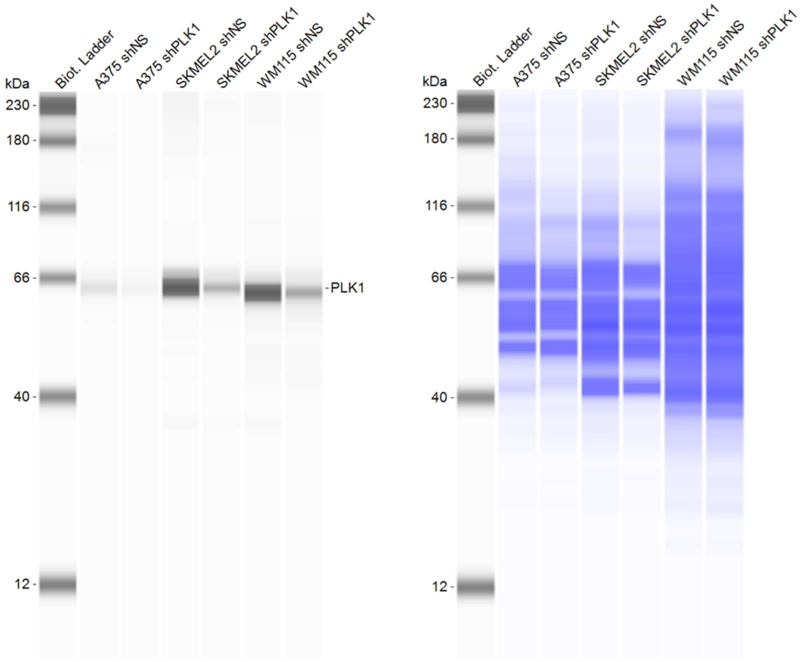

N-cadherin

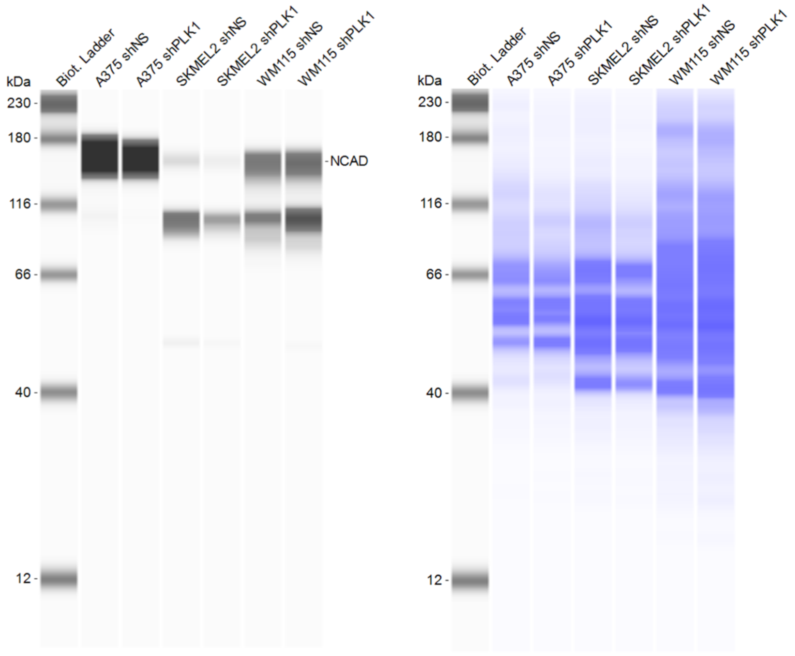

E-cadherin

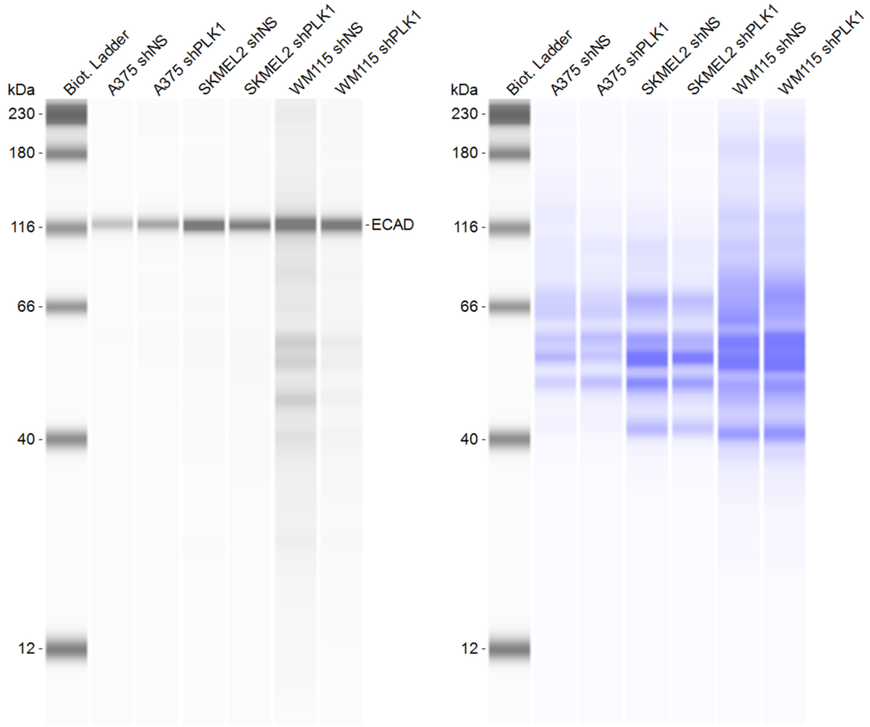

Vimentin

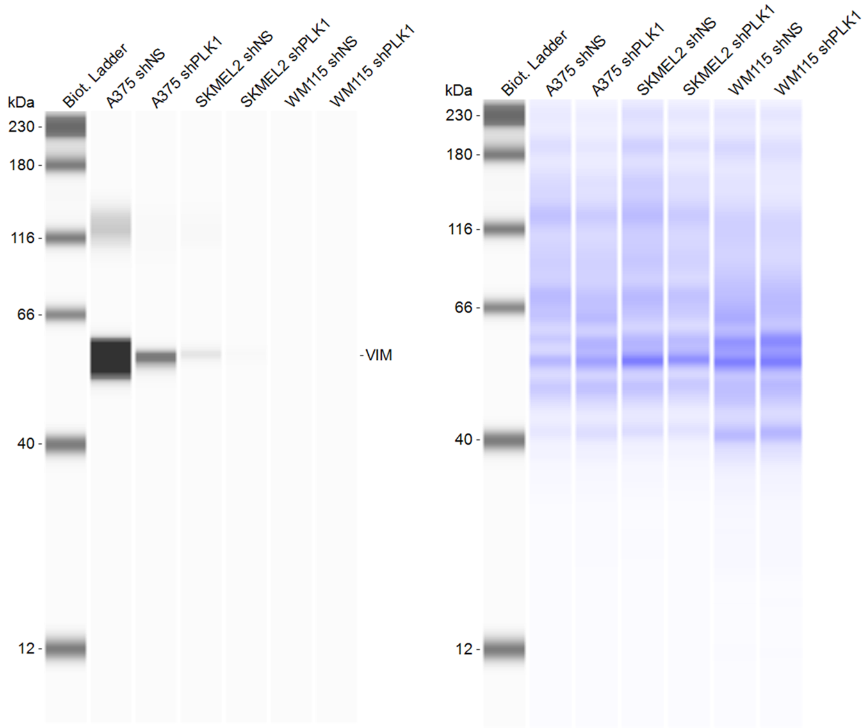

ZEB1

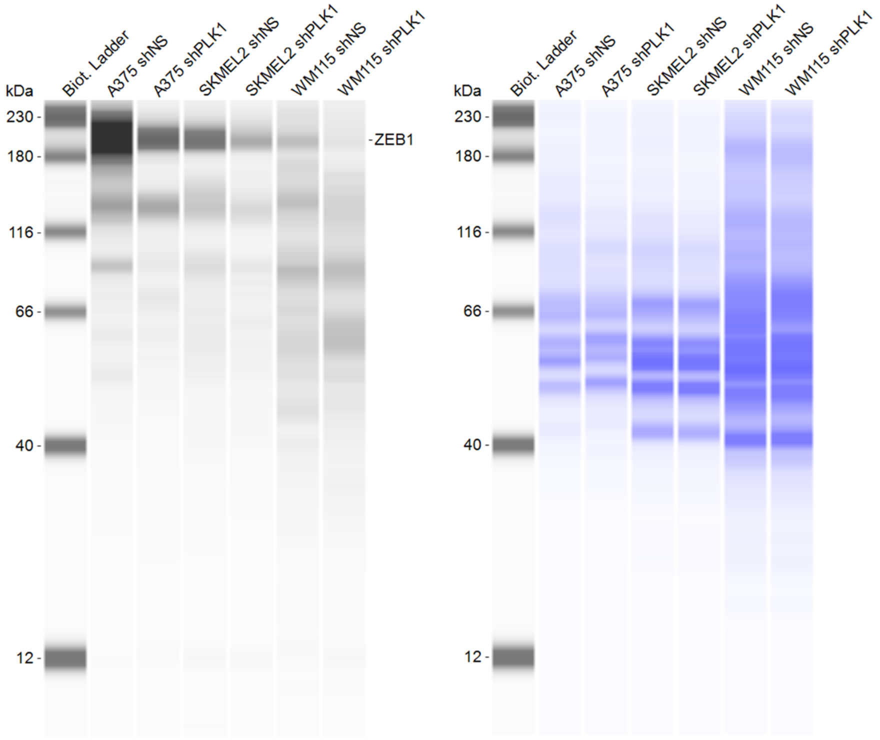

kDa
Biot. Ladder
A375 shNS
A375 shPLK1
SKMEL2 shNS
SKMEL2 shPLK1
WM115 shNS
WM115 shPLK1
230
180
116
66
40
12
ZEB1

SNAIL

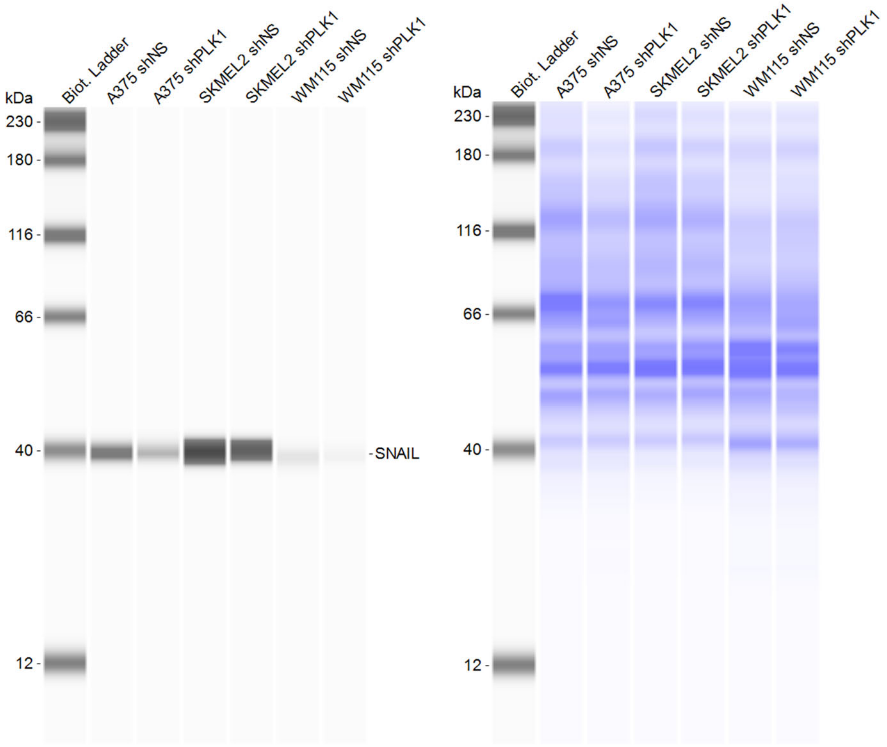

kDa
Biot. Ladder
A375 shNS
A375 shPLK1
SKMEL2 shNS
SKMEL2 shPLK1
WM115 shNS
WM115 shPLK1
230
180
116
66
40
12
SNAIL

Supplementary Figure 8. Uncropped Simple Western immunoblots and total protein assay images for immunoblot data shown in Figure 3.

PLK1

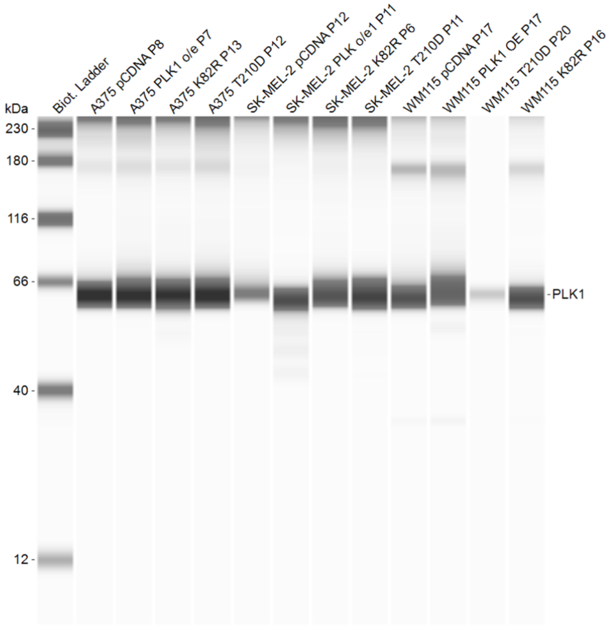

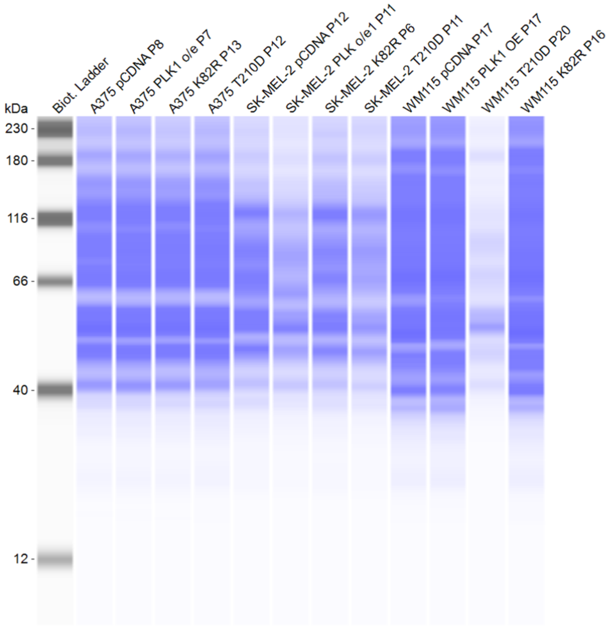

N-cadherin

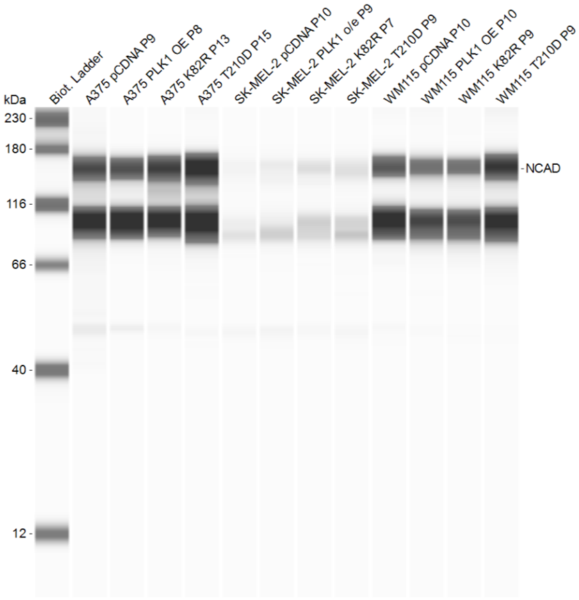

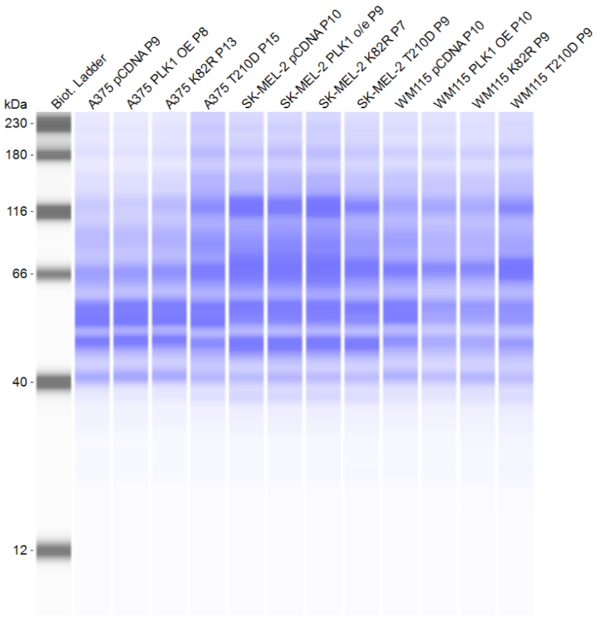

E-cadherin

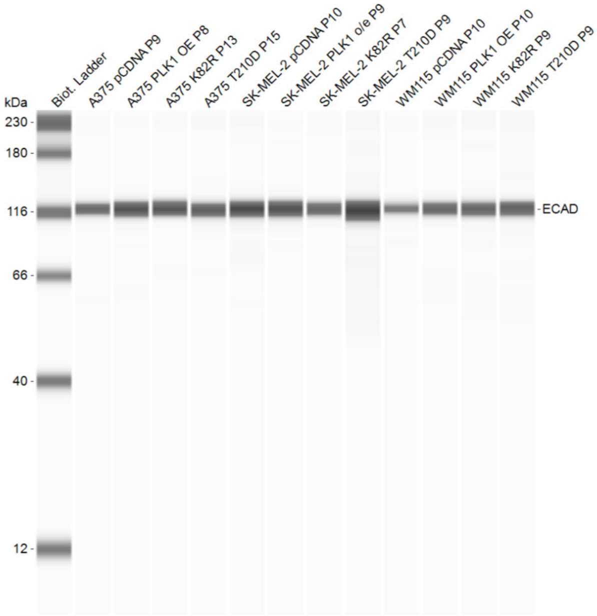

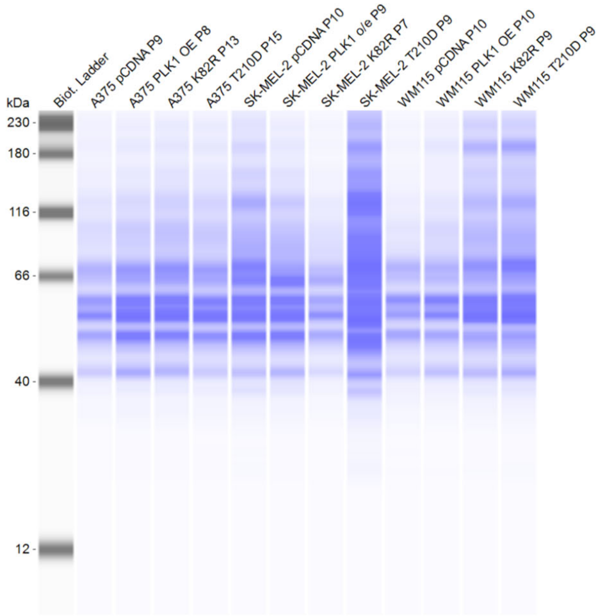

Vimentin

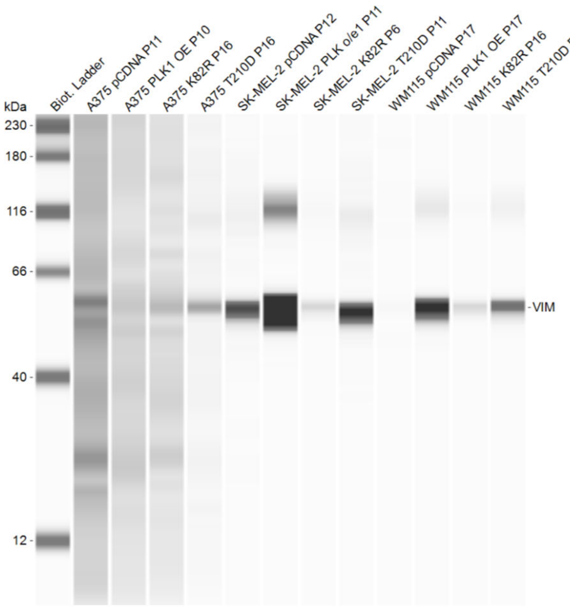

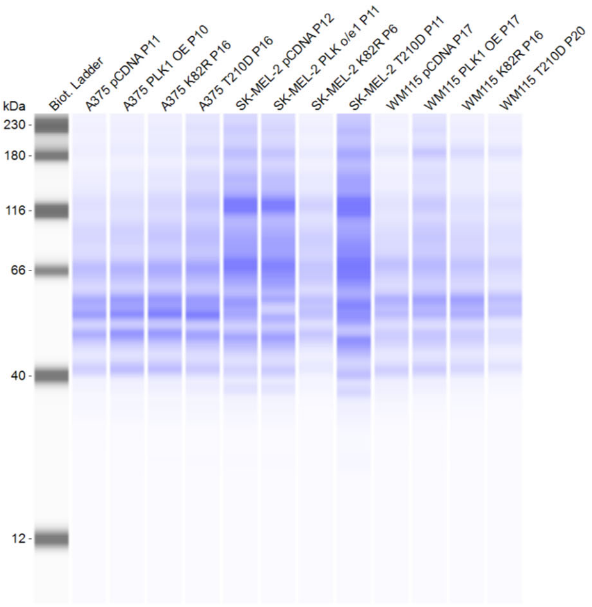

ZEB1

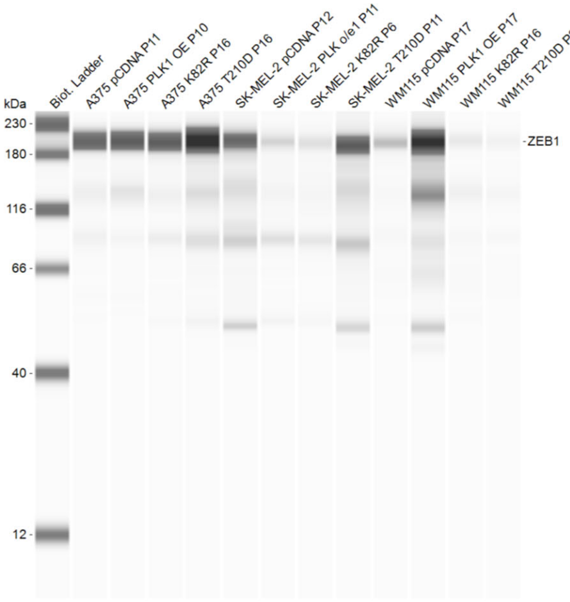
kDa
Biot. Ladder
A375 pCDNA P11
A375 PLK1 OE P10
A375 K82R P16
A375 T210D P16
SK-MEL-2 pCDNA P12
SK-MEL-2 PLK o/e1 P11
SK-MEL-2 K82R P6
SK-MEL-2 T210D P11
WM115 pCDNA P17
WM115 PLK1 OE P17
WM115 K82R P16
WM115 T210D P20
230
180
116
66
40
12
ZEB1

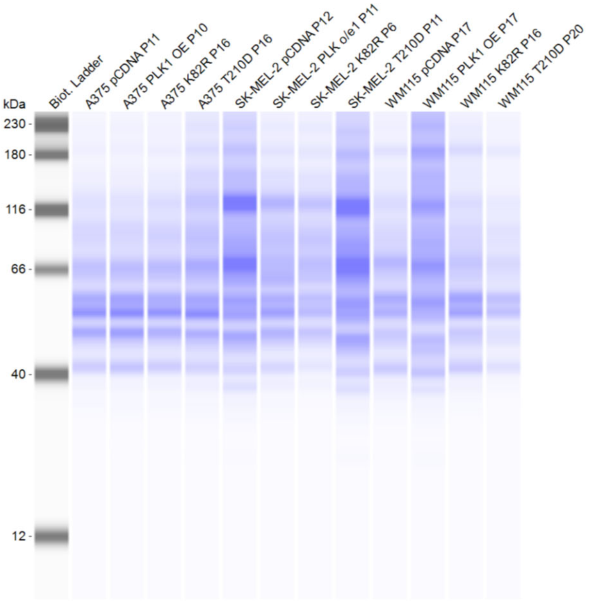
kDa
Biot. Ladder
A375 pCDNA P11
A375 PLK1 OE P10
A375 K82R P16
A375 T210D P16
SK-MEL-2 pCDNA P12
SK-MEL-2 PLK o/e1 P11
SK-MEL-2 K82R P6
SK-MEL-2 T210D P11
WM115 pCDNA P17
WM115 PLK1 OE P17
WM115 K82R P16
WM115 T210D P20
230
180
116
66
40
12

SNAIL

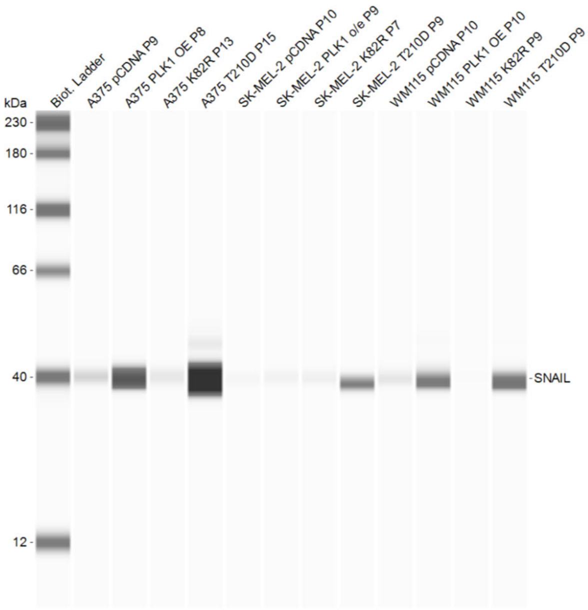
kDa
Biot. Ladder
A375 pCDNA P9
A375 PLK1 OE P8
A375 K82R P13
A375 T210D P15
SK-MEL-2 pCDNA P10
SK-MEL-2 PLK1 o/e P9
SK-MEL-2 K82R P7
SK-MEL-2 T210D P9
WM115 pCDNA P10
WM115 PLK1 OE P10
WM115 K82R P9
WM115 T210D P9
230
180
116
66
40
12
SNAIL

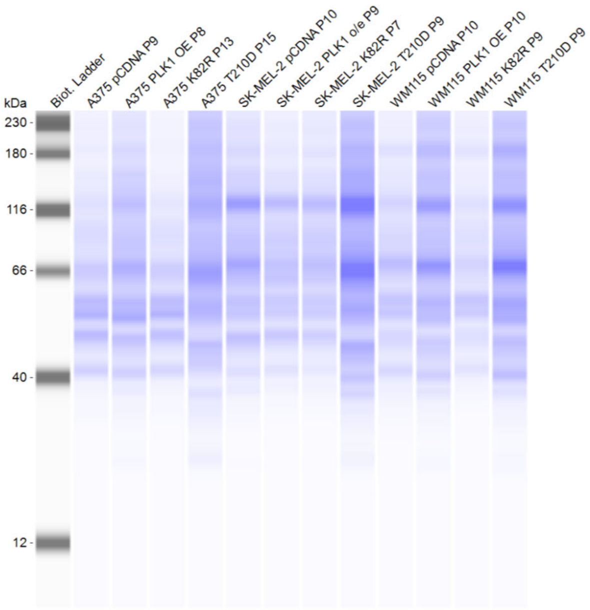
kDa
Biot. Ladder
A375 pCDNA P9
A375 PLK1 OE P8
A375 K82R P13
A375 T210D P15
SK-MEL-2 pCDNA P10
SK-MEL-2 PLK1 o/e P9
SK-MEL-2 K82R P7
SK-MEL-2 T210D P9
WM115 pCDNA P10
WM115 PLK1 OE P10
WM115 K82R P9
WM115 T210D P9
230
180
116
66
40
12

Supplementary Figure 9. Uncropped immunoblots for data shown in Supplementary Figure 3.

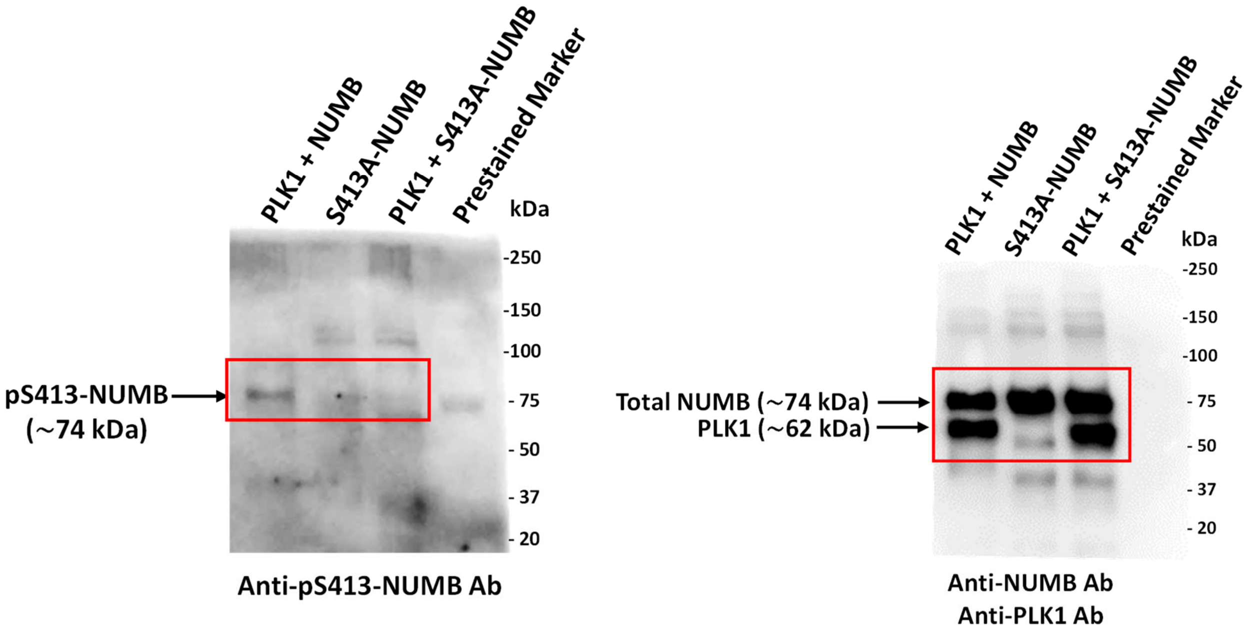

Supplementary Figure 10. Uncropped Simple Western immunoblots and total protein assay images for immunoblot data shown in Figure 6a.

NICD

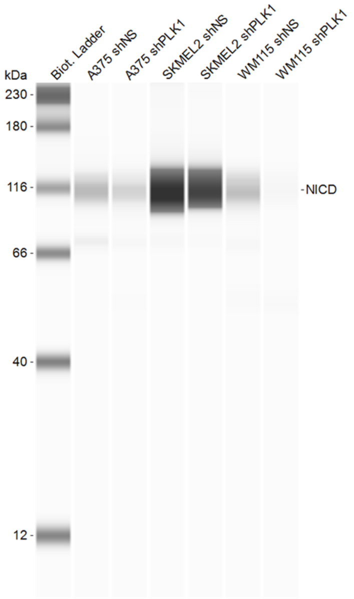

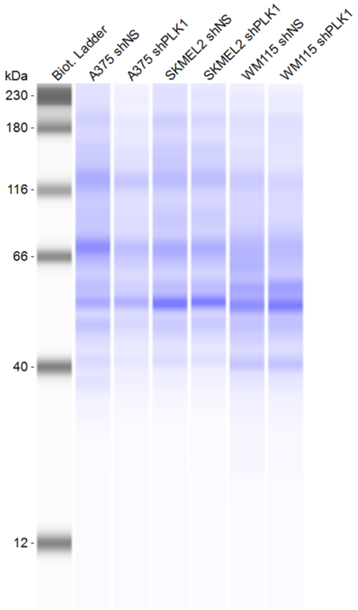

Supplementary Figure 11. Uncropped Simple Western immunoblots and total protein assay images for immunoblot data shown in Figure 6b.

NICD

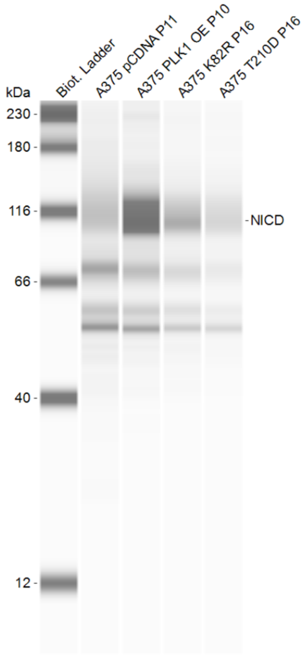

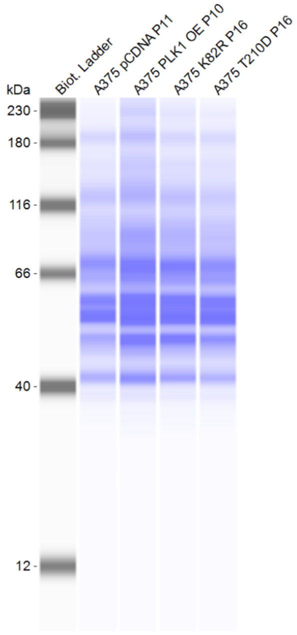

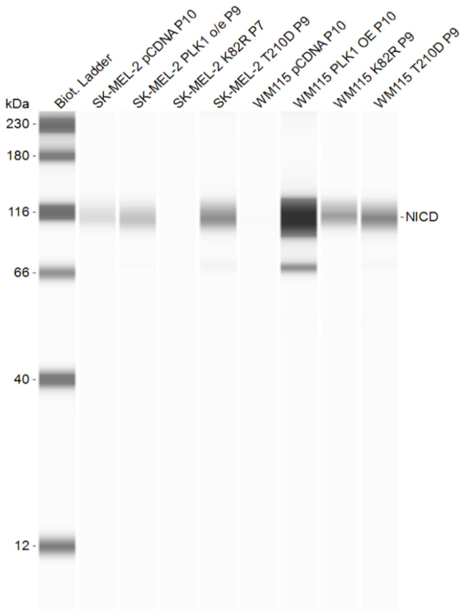

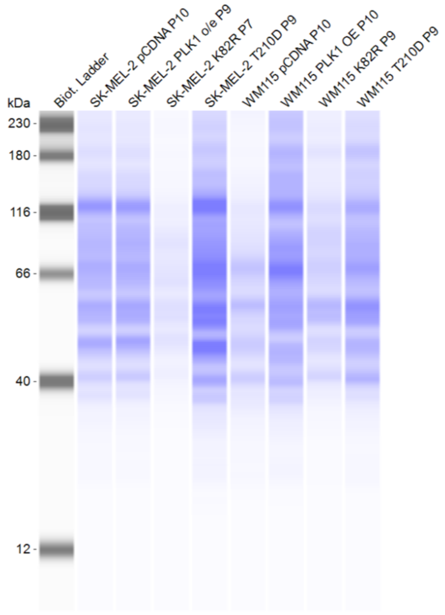

Supplementary Table 1. List of upregulated and downregulated genes identified in response to PLK1 kd at cut-off point >2-fold change with p-value<0.05.

Gene Symbol	Expr Fold Change	Entrez Gene Name	Location	Type(s)
ABLIM3	2.06	actin binding LIM protein family member 3	Cytoplasm	other
ACSL5	2.78	acyl-CoA synthetase long chain family member 5	Cytoplasm	enzyme
ADAM23	-2.4	ADAM metallopeptidase domain 23	Plasma Membrane	peptidase
ADGRF4	2.09	adhesion G protein-coupled receptor F4	Plasma Membrane	G-protein coupled receptor
AGMO	-6.6	alkylglycerol monooxygenase	Cytoplasm	enzyme
AIM2	-2.71	absent in melanoma 2	Cytoplasm	other
AJAP1	2.02	adherens junctions associated protein 1	Plasma Membrane	other
AJUBA	2.3	ajuba LIM protein	Nucleus	transcription regulator
ALPK2	2.91	alpha kinase 2	Nucleus	kinase
ANKRD1	2.25	ankyrin repeat domain 1	Cytoplasm	transcription regulator
ANKRD20A4P	-2.44	ankyrin repeat domain 20 family member A4, pseudogene	Plasma Membrane	other
ANKRD29	2.6	ankyrin repeat domain 29	Other	other
ANKRD30B	2.2	ankyrin repeat domain 30B	Extracellular Space	other
ANXA10	2.28	annexin A10	Cytoplasm	other
AP3S1	2.22	adaptor related protein complex 3 subunit sigma 1	Cytoplasm	transporter
AREG	2.61	amphiregulin	Extracellular Space	growth factor
ARFGEF3	2.11	ARFGEF family member 3	Other	other
ARHGAP29	3.68	Rho GTPase activating protein 29	Cytoplasm	other
ARHGDIB	2.28	Rho GDP dissociation inhibitor beta	Cytoplasm	enzyme
ART3	-2.09	ADP-ribosyltransferase 3 (inactive)	Plasma Membrane	enzyme

AXL	2.17	AXL receptor tyrosine kinase	Plasma Membrane	kinase
BST1	2.11	bone marrow stromal cell antigen 1	Plasma Membrane	enzyme
BTBD11	2.07	BTB domain containing 11	Other	transcription regulator
C18orf54	2.01	chromosome 18 open reading frame 54	Extracellular Space	other
C19orf33	3.03	chromosome 19 open reading frame 33	Nucleus	other
C3	-2.01	complement C3	Extracellular Space	peptidase
CABYR	2.23	calcium binding tyrosine phosphorylation regulated	Cytoplasm	other
CADM1	-3.12	cell adhesion molecule 1	Plasma Membrane	other
CALB2	3.62	calbindin 2	Cytoplasm	other
CBLB	-2.01	Cbl proto-oncogene B	Nucleus	enzyme
CCL2	7.11	C-C motif chemokine ligand 2	Extracellular Space	cytokine
CCNA1	2.08	cyclin A1	Nucleus	other
CD74	-2.13	CD74 molecule	Plasma Membrane	transmembrane receptor
CDCP1	3.55	CUB domain containing protein 1	Plasma Membrane	other
CDH19	-2.78	cadherin 19	Plasma Membrane	other
CEMIP2	-2.1	cell migration inducing hyaluronidase 2	Cytoplasm	enzyme
CFI	-2.33	complement factor I	Extracellular Space	peptidase
CHRDL1	-3	chordin like 1	Extracellular Space	other
CHRNA9	-8.44	cholinergic receptor nicotinic alpha 9 subunit	Plasma Membrane	transmembrane receptor
CLDN10-AS1	-4.51	CLDN10 antisense RNA 1	Other	other
CLEC2B	-2.15	C-type lectin domain family 2 member B	Plasma Membrane	other
CNTNAP3P2	-2.53	CNTNAP3 pseudogene 2	Other	other
COL12A1	2.42	collagen type XII alpha 1 chain	Extracellular Space	other

COLEC12	-3.12	collectin subfamily member 12	Plasma Membrane	transmembrane receptor
CPA4	4.07	carboxypeptidase A4	Extracellular Space	peptidase
CST2	2.27	cystatin SA	Extracellular Space	other
CT55	2.91	cancer/testis antigen 55	Other	other
CTAG2	2.02	cancer/testis antigen 2	Cytoplasm	other
CTNNAL1	2.12	catenin alpha like 1	Plasma Membrane	other
CXADRP3	2.52	CXADR pseudogene 3	Other	other
CYP4F11	2.12	cytochrome P450 family 4 subfamily F member 11	Cytoplasm	enzyme
DCC	2.73	DCC netrin 1 receptor	Plasma Membrane	transmembrane receptor
DOCK11	3.05	dedicator of cytokinesis 11	Cytoplasm	other
DTNA	2.79	dystrobrevin alpha	Plasma Membrane	other
DYNAP	3.08	dynactin associated protein	Other	other
EPG5	2.1	ectopic P-granules autophagy protein 5 homolog	Cytoplasm	other
EPHA2	2.06	EPH receptor A2	Plasma Membrane	kinase
EPHA3	-24.64	EPH receptor A3	Plasma Membrane	kinase
EPHA4	-2.38	EPH receptor A4	Plasma Membrane	kinase
EPHA7	-5.54	EPH receptor A7	Plasma Membrane	kinase
ERBB3	-4.36	erb-b2 receptor tyrosine kinase 3	Plasma Membrane	kinase
EYA1	-3.07	EYA transcriptional coactivator and phosphatase 1	Nucleus	phosphatase
FAM155A	3.18	family with sequence similarity 155 member A	Plasma Membrane	other
FARP1	-2.69	FERM, ARH/RhoGEF and pleckstrin domain protein 1	Plasma Membrane	other
FBXL7	2.14	F-box and leucine rich repeat protein 7	Cytoplasm	enzyme

FLJ46906	-2.53	uncharacterized LOC441172	Other	other
FLRT3	-3.03	fibronectin leucine rich transmembrane protein 3	Plasma Membrane	other
FLT1	-2.28	fms related receptor tyrosine kinase 1	Plasma Membrane	kinase
FOXA1	2.07	forkhead box A1	Nucleus	transcription regulator
FOXO1	-2.34	forkhead box O1	Nucleus	transcription regulator
FOXP1	2.78	forkhead box P1	Nucleus	transcription regulator
FUT9	2.42	fucosyltransferase 9	Cytoplasm	enzyme
FXYD3	-2.91	FXYD domain containing ion transport regulator 3	Plasma Membrane	ion channel
GAGE1	-2.33	G antigen 1	Other	other
GAGE13	-2.42	G antigen 12F	Other	other
GAS7	-2.39	growth arrest specific 7	Cytoplasm	other
GATA6	2.09	GATA binding protein 6	Nucleus	transcription regulator
GBP2	-2.07	guanylate binding protein 2	Cytoplasm	enzyme
GDPD1	-2.12	glycerophosphodiester phosphodiesterase domain containing 1	Cytoplasm	enzyme
GMFG	-2.3	glia maturation factor gamma	Cytoplasm	growth factor
GNG4	-4.6	G protein subunit gamma 4	Plasma Membrane	enzyme
GRAMD1B	-2.05	GRAM domain containing 1B	Cytoplasm	transporter
GRASLND	-2.09	glycosaminoglycan regulatory associated long non-coding RNA	Other	other
GRIP1	-3.32	glutamate receptor interacting protein 1	Plasma Membrane	transcription regulator
H4C8	2.4	H4 clustered histone 8	Nucleus	other
HBEGF	2.04	heparin binding EGF like growth factor	Extracellular Space	growth factor
HCP5	2.04	HLA complex P5	Other	other

HLA-DQA1	-4.69	major histocompatibility complex, class II, DQ alpha 1	Plasma Membrane	transmembrane receptor
HLA-DQB1	-2.05	major histocompatibility complex, class II, DQ beta 1	Plasma Membrane	other
HLA-DRA	-6.55	major histocompatibility complex, class II, DR alpha	Plasma Membrane	transmembrane receptor
HLA-DRB1	-6.41	major histocompatibility complex, class II, DR beta 1	Plasma Membrane	transmembrane receptor
HLA-DRB3	-4.12	major histocompatibility complex, class II, DR beta 3	Cytoplasm	other
HLA-DRB4	-10.21	major histocompatibility complex, class II, DR beta 4	Plasma Membrane	transmembrane receptor
HLA-DRB6	-3.4	major histocompatibility complex, class II, DR beta 6 (pseudogene)	Other	other
HMCN1	-2.8	hemicentin 1	Extracellular Space	other
IGFBP5	-7.88	insulin like growth factor binding protein 5	Extracellular Space	other
IGFBP7	-2.06	insulin like growth factor binding protein 7	Extracellular Space	transporter
IL24	2.08	interleukin 24	Extracellular Space	cytokine
INHBA	-2.01	inhibin subunit beta A	Extracellular Space	growth factor
INPP5F	-2.18	inositol polyphosphate-5-phosphatase F	Plasma Membrane	phosphatase
ITGA1	-2.41	integrin subunit alpha 1	Plasma Membrane	other
ITGB3	-2.8	integrin subunit beta 3	Plasma Membrane	transmembrane receptor
ITGB8	-2.43	integrin subunit beta 8	Plasma Membrane	other
KCNAB1	2.27	potassium voltage-gated channel subfamily A member regulatory beta subunit 1	Plasma Membrane	ion channel
KCTD12	2.19	potassium channel tetramerization domain containing 12	Plasma Membrane	ion channel
KIAA0040	-3.85	KIAA0040	Other	other
KLF5	-2.12	Kruppel like factor 5	Nucleus	transcription regulator

KLHL4	2.12	kelch like family member 4	Cytoplasm	other
KRT80	2.67	keratin 80	Cytoplasm	other
KYNU	-3.21	kynureninase	Cytoplasm	enzyme
LAPTM5	-2.52	lysosomal protein transmembrane 5	Plasma Membrane	other
LEF1	-2.18	lymphoid enhancer binding factor 1	Nucleus	transcription regulator
LINC00460	-2.27	long intergenic non-protein coding RNA 460	Other	other
LINC00473	-2.07	long intergenic non-protein coding RNA 473	Other	other
LINC00622	-4.85	long intergenic non-protein coding RNA 622	Other	other
LINC00707	2.51	long intergenic non-protein coding RNA 707	Other	other
LINC01186	2.88	long intergenic non-protein coding RNA 1186	Other	other
LINC01239	-4.48	long intergenic non-protein coding RNA 1239	Other	other
LINC01363	-2.92	long intergenic non-protein coding RNA 1363	Other	other
LINC01419	-26.15	long intergenic non-protein coding RNA 1419	Other	other
LINC02587	-2.58	long intergenic non-protein coding RNA 2587	Other	other
LMO3	2.21	LIM domain only 3	Nucleus	other
LOC100507516	2.16	uncharacterized LOC100507516	Other	other
LOC642929	-3.84	general transcription factor II, i pseudogene	Other	other
LOC644249	-3.29	ankyrin repeat domain 18A pseudogene	Other	other
LOC728715	-2.53	ovostatin homolog 2	Other	other
LOXL4	2.52	lysyl oxidase like 4	Extracellular Space	enzyme
LRATD2	-2.02	LRAT domain containing 2	Plasma Membrane	other
LRIG3	-3.24	leucine rich repeats and immunoglobulin like domains 3	Extracellular Space	other
LURAP1L	2.72	leucine rich adaptor protein 1 like	Other	other

LY96	3	lymphocyte antigen 96	Plasma Membrane	transmembrane receptor
LYPD3	-2.71	LY6/PLAUR domain containing 3	Plasma Membrane	other
LYZ	-2.09	lysozyme	Extracellular Space	enzyme
MALT1	2.17	MALT1 paracaspase	Cytoplasm	peptidase
MANCR	2.25	mitotically associated long non coding RNA	Other	other
MCF2	-2.01	MCF.2 cell line derived transforming sequence	Cytoplasm	other
MEOX2	-4.93	mesenchyme homeobox 2	Nucleus	transcription regulator
MGP	-4.19	matrix Gla protein	Extracellular Space	other
mir-622	2.07	microRNA 622	Cytoplasm	microRNA
MMP1	-2.15	matrix metallopeptidase 1	Extracellular Space	peptidase
MMP8	-7.81	matrix metallopeptidase 8	Extracellular Space	peptidase
MUC5B	-2.2	mucin 5B, oligomeric mucus/gel-forming	Extracellular Space	peptidase
MYEF2	3.37	myelin expression factor 2	Nucleus	transcription regulator
MYO10	2.34	myosin X	Cytoplasm	enzyme
NAV3	2.4	neuron navigator 3	Nucleus	other
NDP	2.12	norrin cystine knot growth factor NDP	Extracellular Space	growth factor
NEO1	2.01	neogenin 1	Plasma Membrane	transcription regulator
NES	-3.85	nestin	Extracellular Space	other
NID1	-3.32	nidogen 1	Extracellular Space	other
NID2	3.71	nidogen 2	Extracellular Space	other
NR4A2	-2.46	nuclear receptor subfamily 4 group A member 2	Nucleus	ligand-dependent nuclear receptor
NRG1	3.23	neuregulin 1	Plasma Membrane	growth factor
NTS	-5.68	neurotensin	Extracellular Space	other

OR2M3	2.08	olfactory receptor family 2 subfamily M member 3	Plasma Membrane	G-protein coupled receptor
PCDH17	3.46	protocadherin 17	Plasma Membrane	other
PCDH18	-3.16	protocadherin 18	Extracellular Space	other
PCDH7	-2.22	protocadherin 7	Plasma Membrane	other
PCSK1	-3.42	proprotein convertase subtilisin/kexin type 1	Cytoplasm	peptidase
PDCD1LG2	2.03	programmed cell death 1 ligand 2	Plasma Membrane	enzyme
PDE4B	-2.06	phosphodiesterase 4B	Cytoplasm	enzyme
PGM5P2	2.53	phosphoglucomutase 5 pseudogene 2	Other	other
PKP2	2.9	plakophilin 2	Plasma Membrane	other
PLP1	-2.11	proteolipid protein 1	Plasma Membrane	other
PLXDC2	-4.03	plexin domain containing 2	Extracellular Space	other
PRELID3B	-2.06	PRELI domain containing 3B	Cytoplasm	other
PRRX1	-3.13	paired related homeobox 1	Nucleus	transcription regulator
PTGES	-3.81	prostaglandin E synthase	Cytoplasm	enzyme
PTGS2	-11.25	prostaglandin-endoperoxide synthase 2	Cytoplasm	enzyme
PTPRD	2.33	protein tyrosine phosphatase receptor type D	Plasma Membrane	phosphatase
PTX3	2.02	pentraxin 3	Extracellular Space	other
PURPL	-2.46	p53 upregulated regulator of p53 levels	Other	other
PXYLP1	-3.08	2-phosphoxylose phosphatase 1	Cytoplasm	phosphatase
RAB27B	2.09	RAB27B, member RAS oncogene family	Cytoplasm	enzyme
RAB8B	2.26	RAB8B, member RAS oncogene family	Cytoplasm	enzyme
RNF157	-2.82	ring finger protein 157	Cytoplasm	enzyme

RNVU1-21	-2.19	RNA, variant U1 small nuclear 21	Other	other
RNVU1-22	-2.41		Other	other
RORB	-2.47	RAR related orphan receptor B	Nucleus	ligand-dependent nuclear receptor
RTL1	2.02	retrotransposon Gag like 1	Other	other
S100A4	-2.84	S100 calcium binding protein A4	Cytoplasm	other
SALL1	-2.13	spalt like transcription factor 1	Nucleus	transcription regulator
SAMD9L	2.93	sterile alpha motif domain containing 9 like	Extracellular Space	other
SEMA3D	4.75	semaphorin 3D	Extracellular Space	other
SEMA6A	-2.13	semaphorin 6A	Plasma Membrane	transmembrane receptor
SERPINA3	-11.01	serpin family A member 3	Extracellular Space	other
SERPINB7	2.25	serpin family B member 7	Cytoplasm	other
SFTA1P	2.89	surfactant associated 1, lncRNA	Other	other
SH3GL2	3.95	SH3 domain containing GRB2 like 2, endophilin A1	Plasma Membrane	enzyme
SHC4	-2.29	SHC adaptor protein 4	Cytoplasm	other
SLC14A1	2.67	solute carrier family 14 member 1 (Kidd blood group)	Plasma Membrane	transporter
SLC14A2	2.09	solute carrier family 14 member 2	Plasma Membrane	transporter
SLC16A6	-2.39	solute carrier family 16 member 6	Plasma Membrane	transporter
SLC16A6P1	-2.18	SLC16A6 pseudogene 1	Other	other
SLC1A1	-3.48	solute carrier family 1 member 1	Plasma Membrane	transporter
SLC26A2	-3.48	solute carrier family 26 member 2	Plasma Membrane	transporter
SLC35F1	-2.55	solute carrier family 35 member F1	Other	other

SLITRK6	-6.33	SLIT and NTRK like family member 6	Plasma Membrane	other
SNAI1	-2.19	snail family transcriptional repressor 1	Nucleus	transcription regulator
SNORD15A	-2.2	small nucleolar RNA, C/D box 15A	Other	other
SORCS1	-5.8	sortilin related VPS10 domain containing receptor 1	Plasma Membrane	transporter
SORL1	2.33	sortilin related receptor 1	Cytoplasm	transporter
SOX2	-3.32	SRY-box transcription factor 2	Nucleus	transcription regulator
SOX4	-2.16	SRY-box transcription factor 4	Nucleus	transcription regulator
SOX5	-5.75	SRY-box transcription factor 5	Nucleus	transcription regulator
SOX9	2.35	SRY-box transcription factor 9	Nucleus	transcription regulator
SPANXB1	2.22	SPANX family member C	Nucleus	other
SPP1	-2.97	secreted phosphoprotein 1	Extracellular Space	cytokine
SPRY2	-3.01	sprouty RTK signaling antagonist 2	Plasma Membrane	other
SPTLC3	2.63	serine palmitoyltransferase long chain base subunit 3	Cytoplasm	enzyme
SRGN	3.45	serglycin	Cytoplasm	other
ST8SIA4	-2.08	ST8 alpha-N-acetyl-neuraminide alpha-2,8-sialyltransferase 4	Cytoplasm	enzyme
STK32B	-2.18	serine/threonine kinase 32B	Other	kinase
SULF1	-2.05	sulfatase 1	Cytoplasm	enzyme
SYTL5	-12.1	synaptotagmin like 5	Extracellular Space	other
TENM1	-3.29	teneurin transmembrane protein 1	Plasma Membrane	transmembrane receptor
TENM2	-6.31	teneurin transmembrane protein 2	Plasma Membrane	other

TFAP2B	-6.02	transcription factor AP-2 beta	Nucleus	transcription regulator
TGM2	2.15	transglutaminase 2	Cytoplasm	enzyme
TIMP3	-2.17	TIMP metallopeptidase inhibitor 3	Extracellular Space	other
TMEM200A	2.2	transmembrane protein 200A	Other	other
TNFRSF19	-2.69	TNF receptor superfamily member 19	Plasma Membrane	transmembrane receptor
TRIML2	2.06	tripartite motif family like 2	Other	other
TRPA1	-2.15	transient receptor potential cation channel subfamily A member 1	Plasma Membrane	transporter
TRPC4	4.61	transient receptor potential cation channel subfamily C member 4	Plasma Membrane	ion channel
TSHZ2	-4.47	teashirt zinc finger homeobox 2	Nucleus	transcription regulator
TSPAN13	-4.49	tetraspanin 13	Plasma Membrane	other
VEPH1	2.02	ventricular zone expressed PH domain containing 1	Nucleus	other
ZHX2	2.5	zinc fingers and homeoboxes 2	Nucleus	transcription regulator
ZNF280A	2.25	zinc finger protein 280A	Nucleus	transcription regulator
ZNF385D-AS1	-7.68	ZNF385D antisense RNA 1	Other	other
ZNF521	-2.83	zinc finger protein 521	Nucleus	transcription regulator
ZNF716	2.18	zinc finger protein 716	Other	other

Supplementary Table 2. Imaging and analysis settings for NICD nuclear localization using the BioTek Lionheart FL.

Primary Object Selection		Secondary Mask Selection	
Parameter	Value	Parameter	Value
Channel	DAPI 377,447	Channel	GFP 469,525
**Threshold**		Background	Dark
Value	5000	Measure Within a Primary Mask	Yes
Background	Dark	Use Primary Mask	Yes
Split Touching Object	Yes	Measure Within a Secondary Mask	No
Fill Holes in Masks	Yes		
**Advanced Options**		**Calculated Metrics**	
Background Flattening		Data	Calculate?
Rolling Ball	Auto	Cell count	Yes
Smoothing	1	Object Size	Yes
Background	5% of lowest pixels	Object Area	Yes
Object Size Selection		Object Sum Area	Yes
Minimum	5	Object Mean[DAPI 377,447]	Yes
Maximum	100	Object Area[GFP 469,525]	Yes
Include Edge Objects	No	Object Sum Area[GFP 469,525]	Yes
Entire Image	Yes	Object Mean[GFP 469,525]	Yes
